# Microstructural Changes in the Corpus Callosum in Different Forms of Sporadic Age-Related Cerebral Small Vessel Disease

**DOI:** 10.3390/diagnostics16172861

**Published:** 2026-09-05

**Authors:** Elena I. Kremneva, Larisa A. Dobrynina, Kamila V. Shamtieva, Anastasia A. Geints, Mikhail S. Sokolov, Maryam R. Zabitova, Alexey S. Filatov, Marina V. Krotenkova

**Affiliations:** 1 Russian Center of Neurology and Neurosciences, 125367 Moscow, Russia; moomin10j@mail.ru (E.I.K.); dobrla@mail.ru (L.A.D.); kamila.shamt@gmail.com (K.V.S.); anastasiyatarasova75@gmail.com (A.A.G.); filatov.a.s@neurology.ru (A.S.F.); krotenkova_mrt@mail.ru (M.V.K.); 2 Soft Matter and Physics of Fluids Centre, Bauman Moscow State Technical University, 105005 Moscow, Russia; toptyginen@gmail.com

**Keywords:** cerebral small vessel disease, diffusion MRI, white matter microstructure, MC-SMT, corpus callosum, cognitive impairment, heterogeneity

## Abstract

**Background/Objectives:** Cerebral small vessel disease (SVD) is a heterogeneous condition in which similar conventional MRI findings may be associated with different clinical manifestations and pathogenetic mechanisms. Previously, hierarchical clustering of structural MRI features in patients with severe white matter hyperintensities (Fazekas 3) identified two MRI phenotypes, designated MRI Type 1 and MRI Type 2. Diffusion MRI (dMRI) may provide additional information about the microstructural differences between these phenotypes. To compare white matter microstructure between MRI Type 1 and MRI Type 2 of sporadic age-related SVD using signal-based and biophysical dMRI models. **Methods**: This cross-sectional study included 75 patients with SVD and 36 age- and sex-matched healthy controls. Among the patients with SVD, 43 had MRI Type 1 and 32 had MRI Type 2. All participants underwent structural and multi-shell dMRI on a 3 Tesla MRI scanner. Diffusion metrics were derived using multiple models: Diffusion Tensor Imaging (DTI), Diffusion Kurtosis Imaging (DKI), Neurite Orientation Dispersion and Density Imaging (NODDI), White Matter Tract Integrity (WMTI), and the Multi-compartment Spherical Mean Technique (MC-SMT). Tract-profile analysis was performed in three corpus callosum segments: the forceps major, forceps minor, and body. Group differences were assessed using age- and sex-adjusted general linear models with correction for multiple comparisons. The combined discriminative value of dMRI metrics was evaluated using regularized Elastic Net logistic regression with repeated nested five-fold cross-validation. **Results:** After adjustment for age and sex, the overall group effect remained significant for 45 of 48 global dMRI measures following Benjamini–Hochberg correction. Compared with MRI Type 2, MRI Type 1 showed lower fractional anisotropy (FA), neurite density index (NDI), intra-axonal volume fraction (INTRA), axonal water fraction (AWF), mean kurtosis (MK), axial kurtosis (AK), and radial kurtosis (RK), and higher mean diffusivity (MD), radial diffusivity (RD), extra-axonal mean diffusivity (EXTRA_MD), extra-axonal transverse diffusivity (EXTRA_TRANS), and extra-axonal radial diffusivity (radEAD). These differences were generally most pronounced in the body of the corpus callosum. In the segmental analysis, 131 of 144 values showed a significant overall group effect after correction, and 108 demonstrated significant differences between MRI Type 1 and MRI Type 2. The largest effects were observed in the 60–80% interval of the corpus callosum body, particularly for AWF, MK, INTRA, EXTRA_TRANS, RK, FA, RD, radEAD, and MD. An Elastic Net model combining age, sex, and 48 global dMRI measures discriminated MRI Type 1 from MRI Type 2 with an internally validated area under the curve of 0.866 (95% CI, 0.762–0.953), accuracy of 86.7%, sensitivity of 75.0%, and specificity of 95.3%. Ten dMRI features showed a selection frequency of at least 70% across repeated model construction. **Conclusions:** MRI Type 1 is characterized by more severe and spatially extensive corpus callosum microstructural abnormalities than MRI Type 2, despite broadly similar vascular risk-factor profiles. The findings support the heterogeneity of sporadic age-related SVD and indicate that combined signal-based and biophysical dMRI metrics may improve MRI phenotyping. The observed associations should be interpreted as indirect markers of tissue microstructure and require confirmation in larger, independent, and longitudinal cohorts.

## 1. Introduction

Age-related (sporadic) cerebral small vessel disease (SVD) is a highly prevalent and disabling condition [1,2]. According to multicenter epidemiological and pathological studies, it is the leading cause of isolated vascular and mixed neurodegenerative-vascular cognitive impairment (CI), contributing to at least 45% of newly registered dementia cases annually, 20–25% of ischemic strokes, and the majority of hemorrhagic strokes [3,4,5,6].

In recent years there has been an unprecedented rise in interest in this problem, driven by the recognition that controlling its main risk factor, the arterial hypertension (AH), alone is insufficient to combat the disease. Clinico-radiological comparisons indicate the absence of a direct causal relationship between the severity of AH and the clinical and MRI manifestations of SVD. One explanation for this discrepancy may be the heterogeneity of the disease forms, whose development is associated with the predominance of different pathogenetic mechanisms [7,8,9]. Until recently, hypoxia and ischemia resulting from progressive arteriolosclerosis were considered the only mechanism of brain damage in SVD [10,11,12]. However, over the past decades, increasing evidence points to endothelial dysfunction and chronic sterile inflammation—which trigger blood–brain barrier (BBB) damage and neuroinflammation—as contributors to the initiation and progression of the disease [13,14,15,16]. Contemporary understanding of the pathogenesis of SVD also emphasizes the role of glymphatic dysfunction and impaired interstitial clearance [17,18,19].

The original approach we previously used to clarify these heterogeneous forms was based on grouping (clustering) MRI features (white matter hyperintensity (WMH), lacunes, enlarged perivascular spaces (PVS), cerebral microbleeds (CMB), and brain atrophy) according to their severity and location. Such approach allowed us to identify two clusters of features in Fazekas stage 3 WMH [20], which we designated as MRI Type 1 and Type 2. These did not differ in the severity of vascular risk factors, but differed in the severity of clinical manifestations and, accordingly, in disease prognosis. Associations established in subsequent studies between MRI Type 1 and decreased vascular endothelial growth factor-A (VEGF-A), and between MRI Type 2 and increased tumor necrosis factor-alpha (TNF-α) and increased BBB permeability on T1-dynamic contrast-enhanced MRI suggested a potentially greater contribution of ischemic/hypoxic mechanisms and endothelial injury to MRI Type 1, and of BBB dysfunction and inflammation to MRI Type 2 [21,22,23,24,25].

The use of diffusion MRI (dMRI) can substantially contribute to differentiating the heterogeneous forms of SVD based on the pattern and severity of microstructural changes. Its high sensitivity to microstructural changes in visually unaffected (normal-appearing) white matter has previously been used successfully to identify diffusion-based equivalents of SVD severity and associated cognitive impairment (CI) [26,27].

The dMRI method is based on assessing the predominant direction of water molecule movement within a brain tissue voxel. Conventional signal models (e.g., diffusion tensor MRI) represent this in aggregate (usually for white or gray matter as a whole), whereas biophysical models represent it within conditional tissue compartments (intra-axonal, extra-axonal in different directions—typically transverse and longitudinal) [28]. Thus, the use of metrics from both signal and biophysical models may help clarify the microstructural differences underlying the MRI types, which reflect the severity and preferential localization of brain and compartment damage that give rise to the heterogeneous forms of the disease.

The aim of the study was to evaluate the microstructural changes in the two forms of SVD diagnosed by clustering (grouping) of its features—MRI Type 1 and Type 2.

## 2. Materials and Methods

The study was conducted at the Russian Center of Neurology and Neurosciences (Moscow) between 2016 and 2022, on the basis of the Department of Radiology and the 3rd Neurological Department. The study was approved by the local Ethics Committee of the Russian Center of Neurology and Neurosciences (Protocol No. 2-4/16, dated 17 February 2016). All subjects signed informed consent to participate in the study.

The initial SVD cohort comprised 114 patients. Of these, 75 patients had complete analyzable multi-shell dMRI data and were included in the present dMRI analysis, whereas 39 patients did not have complete analyzable multi-shell dMRI data and were therefore not included in this analysis. To assess the possibility of selection bias, baseline demographic and clinical characteristics were compared between patients included and not included in the dMRI analysis. The results of this comparison are presented in Supplementary Table S1.

**Figure 1:**
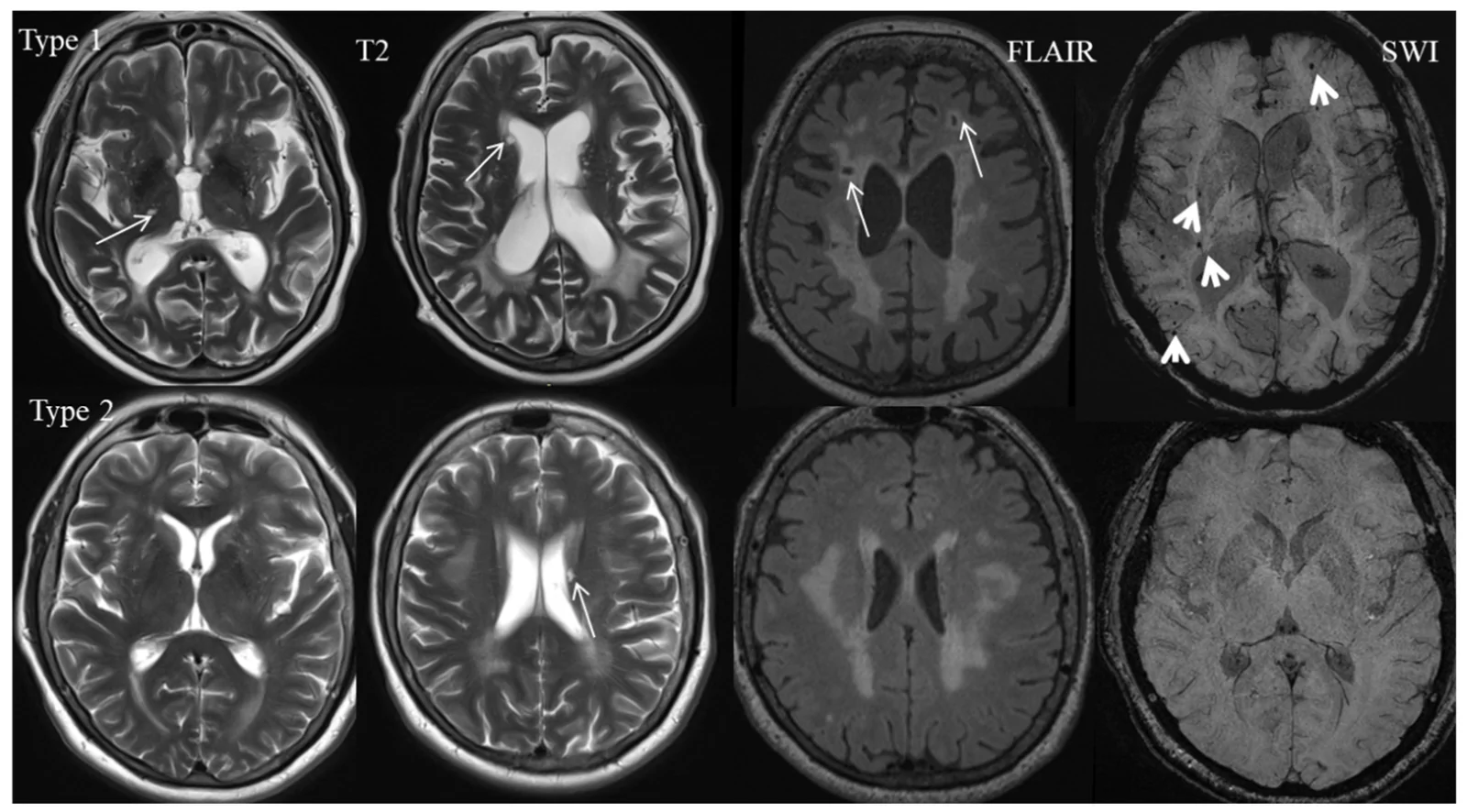
Examples of brain MRI in patients with structural changes characteristic of MRI Type 1 (**upper row**) and MRI Type 2 (**lower row**). Thin arrows—lacunes; short arrows—cerebral microbleeds.

The MRI types corresponded to the 2 clusters obtained by hierarchical analysis of all SVD MRI features. These features were scored on a 4-point severity scale separately for juxtacortical, deep, and periventricular white matter and subcortical structures in each brain lobe [20]. The clustering incorporated the combined severity and spatial distribution of structural SVD MRI features, including white matter hyperintensities, lacunes, cerebral microbleeds, brain atrophy, and enlarged perivascular spaces. The principal MRI characteristics of the resulting clusters are summarized in Supplementary Table S6. Examples of the patterns of structural brain changes characteristic of each SVD form (MRI Type) are shown in Figure 1.

In brief, MRI Type 1 was characterized by a higher structural SVD burden, with predominantly confluent periventricular and deep white matter hyperintensities in the frontal and parietal regions extending into the deep occipital white matter, multiple lacunes, juxtacortical and subcortical cerebral microbleeds, more prominent brain atrophy, and more pronounced enlarged perivascular spaces in subcortical structures. MRI Type 2 was characterized by predominantly posterior periventricular white matter hyperintensities with deep frontal and parietal involvement, fewer lacunes, absence of juxtacortical and subcortical cerebral microbleeds, and less prominent/absent atrophy.

Both MRI types were represented among the patients included in the present study.

Inclusion criteria: age 46 to 75 years; MRI changes meeting the STRIVE criteria for SVD (2013, 2023) [29,30]; presence of cognitive complaints.

Exclusion criteria: probable Alzheimer’s disease [31]; other forms of SVD (inflammatory, toxic, thrombophilic, systemic, genetic; history of severe migraine); MRI changes other than SVD; atherosclerotic stenosis of extra- or intracranial arteries >50%; severe decompensated somatic disease; contraindications to MRI; cognitive impairment of such severity that it precluded adequate understanding and following of study procedures, the ability to remain sufficiently still during MRI acquisition, or adequate completion of the examination protocol. Given the sensitivity of multi-shell dMRI to motion-related artifacts, this criterion primarily applied to patients with more severe cognitive impairment who were unable to complete the required MRI acquisition. Dementia itself was not an exclusion criterion if the patient was able to understand the study instructions and complete the MRI protocol.

The clinical work-up included: collection of medical history regarding disease course and prior illnesses; assessment of the main vascular risk factors (AH and its severity, diabetes mellitus, smoking, hypercholesterolemia, obesity, alcohol abuse); general physical examination and assessment of somatic status; and assessment of neurological and cognitive status using the Montreal Cognitive Assessment (MoCA) scale. Cognitive status was categorized taking into account both the MoCA score and functional independence in everyday activities. Subjective cognitive impairment (SubCI) was defined by the presence of cognitive complaints without objective cognitive impairment (MoCA score ≥ 26). Mild cognitive impairment (MCI) was defined as a MoCA score < 26 with preserved independence according to the Instrumental Activities of Daily Living (IADL) assessment, whereas dementia was defined by cognitive impairment accompanied by impairment of independence in everyday activities.

The control group for the dMRI analysis comprised 36 age- and sex-matched healthy volunteers.

Brain MRI was performed on a 3 Tesla Siemens Magnetom Verio scanner (Siemens AG, Siemens Healthineers, Erlangen, Germany). All subjects underwent a standard MRI protocol, followed by assessment of STRIVE features according to the method developed at the Russian Center of Neurology and Neurosciences [20] for features clustering. dMRI—a spin-echo echo-planar sequence in the axial plane (parallel to the line connecting the anterior and posterior commissures) with three b-values (0, 1000, and 2500 s/mm^2^) for 64 diffusion-encoding gradient directions, a 2 × 2 × 2 mm cubic voxel, TR 12,600 ms, TE 115 ms. The scanning protocol also included an additional sequence with four repeats at b = 0 s/mm^2^ and identical parameters, except for the opposite phase-encoding direction (posterior-to-anterior).

Preprocessing of the entire diffusion dataset was performed using an optimized pipeline [32]. This included noise estimation and correction using Marchenko–Pastur principal component analysis of the noise distribution, as well as correction of artifacts related to incomplete k-space sampling (the Gibbs phenomenon). Static magnetic field inhomogeneity was corrected using the FMRIB Software Library (FSL) version 6.0.7. (Department of Clinical Neurosciences, University of Oxford, UK) utilities “topup” and “eddy”. Geometric distortions related to gradient switching were also corrected, together with spatial misalignment of the images. To improve the numerical stability of the measured parameters, image smoothing was applied using a Gaussian filter with a 1 mm^3^ kernel. Brain volume was automatically extracted to obtain a skull-stripped mask. Each mask was visually inspected and, if necessary, manually corrected in ITK-SNAP (http://www.itksnap.org, assessed on 13 July 2026, PICSL, University of Pennsylvania, USA). Diffusion metric maps were generated using in-house scripts based on MATLAB R2017a (MathWorks, Natick, MA, USA), with a sequential implementation of the steps described in Maximov I.I. et al. (2019) [32]. Maps of the main metrics were obtained for diffusion tensor imaging (DTI), diffusion kurtosis imaging (DKI), NODDI (Neurite Orientation Dispersion and Density Imaging), WMTI (White Matter Tract Integrity), and MC-SMT (Multi-Compartment Spherical Mean Technique). Diffusivity metrics AD, MD, RD, axEAD, radEAD EXTRA_MD and EXTRA_TRANS were expressed in ×10^−3^ mm^2^/s; the remaining metrics were dimensionless.

FA maps were then spatially normalized to MNI space using FSL-based software [33], and were subsequently used to normalize the maps of other diffusion metrics to enable group analysis and comparison.

Corpus callosum tract-profile analysis was performed on the resulting maps using a Python 3 interpreter and modified Dipy scripts (DIPY—Diffusion Imaging In Python) (https://dipy.org, assessed on 13 July 2026) [34] (the source code of the scripts and their modifications is available on https://github.com/mikhail-matrosov/classibundler, assessed on 13 July 2026). This analysis comprised the following steps: (1) calculation of the predominant diffusion direction in each voxel using the orientation distribution function, followed by thresholding of the generalized fractional anisotropy at 0.1; (2) whole-brain tractogram construction using the EuDX algorithm; (3) construction of corpus callosum “centroids” for its three regions—the forceps major, forceps minor, and body—which were matched to the “centroids” of the HCP842 atlas (in MNI stereotactic space); (4) segmentation with a modified RecoBundles algorithm, which clusters streamlines and matches them to atlas bundle centroids within a maximum allowable distance defined by the minimum average direct-flip parameter. This distance threshold restricted the segmented tract to streamlines geometrically consistent with the atlas bundle, limiting inclusion of spurious or anatomically implausible fibers; (5) construction of tract profiles. Each fiber was divided into 100 nodes (0 corresponding to the start of the tract and 100 to its end), at each of which the values of the metrics under study were measured; these data were used to build profiles reflecting the metric values along the entire length of the tract [35]. Node-wise tract profiles were used for descriptive visualization of the spatial distribution of dMRI metrics along the tract. Point-wise between-group comparisons generated as part of the tract-profile visualization were considered exploratory and were not used for confirmatory statistical inference. Confirmatory spatially resolved analysis was performed using three non-overlapping tract intervals (20–40%, 40–60%, and 60–80%) with age- and sex-adjusted general linear models and correction for multiple comparisons. Interval-level rather than node-wise analyses formed the basis of the inferential conclusions.

### Statistical Analysis

Quantitative data were presented as mean and standard deviation or as median and interquartile range, depending on the distribution; categorical data were presented as absolute values and proportions. All tests were two-tailed, with a statistical significance level of 0.05. Given the differences between MRI Type 1 and MRI Type 2 in age and sex, a general linear model was constructed for each dMRI metric, adjusting for these factors, while group membership served as the main factor. Effect size was assessed using partial η^2^.

The primary analysis was performed on the mean metric values across the 5–95% segment of tract length. A total of 48 values were evaluated—16 metrics across 3 segments of the corpus callosum. Additionally, these 48 values were analyzed within the 20–40%, 40–60%, and 60–80% intervals of each of the three corpus callosum segments, yielding 144 segmental parameters. Multiple-testing correction was performed separately for the global and spatially resolved analyses. For the global analysis, the Benjamini–Hochberg procedure was applied across the 48 omnibus group tests (16 dMRI metrics × 3 corpus callosum regions). For the spatially resolved analysis, the Benjamini–Hochberg procedure was applied separately across the 144 omnibus group tests (16 metrics × 3 corpus callosum regions × 3 tract intervals). Thus, the 48 global tests and the 144 segmental tests were treated as two separate families of hypotheses. For parameters retaining a statistically significant overall group effect after FDR correction, three pairwise comparisons were subsequently performed within the corresponding model (MRI Type 1 vs. MRI Type 2, MRI Type 1 vs. control, and MRI Type 2 vs. control), with Bonferroni correction for these three comparisons. For each pairwise contrast, the age- and sex-adjusted between-group difference and its 95% CI were estimated.

For multivariate discrimination between MRI Type 1 and MRI Type 2, regularized Elastic Net logistic regression was used, incorporating age, sex, and the 48 global dMRI values. To assess the additional contribution of the dMRI values, the discriminative performance of the resulting model was compared with a reference model including only age and sex.

Internal model validation and the stability of dMRI feature selection were assessed using repeated nested 5-fold cross-validation: the outer partitioning of the sample into five folds was repeated 10 times, and Elastic Net hyperparameter tuning was performed within an inner 5-fold loop using the training data only.

To prevent information leakage, predictor standardization was performed within each cross-validation training fold and then applied to the corresponding held-out fold. Hyperparameter tuning was restricted to the inner training folds, and the corresponding outer test fold was used exclusively to obtain out-of-fold predictions and was not involved in preprocessing, hyperparameter tuning, or model fitting.

The discriminative performance of the model was assessed by AUC on out-of-fold predictions, with 95% CIs calculated using bootstrap resampling based on 2000 iterations. Classification was based on a fixed probability threshold of 0.50 applied to the out-of-fold predicted probabilities. Sensitivity, specificity, and overall accuracy were also calculated, with 95% CIs obtained by bootstrap resampling based on 2000 iterations. Calibration was assessed on the out-of-fold predictions using the Brier score, calibration intercept, and calibration slope. The stability of dMRI feature selection was assessed by the frequency with which features retained a non-zero coefficient across repeated model construction.

## 3. Results

### 3.1. Clinical Assessment and Risk Factors

The diffusion MRI analysis included 75 patients with SVD (43 patients with MRI Type 1, 32 patients with MRI Type 2) (Table 1).

Patients with the two MRI types differed statistically significantly in age (*p* < 0.001), sex (*p* = 0.002), and severity of cognitive impairment (*p* < 0.001). The groups did not differ in the presence or severity of vascular risk factors, and alcohol abuse was not observed in either group.

Patients included in the dMRI analysis did not differ significantly from the 39 patients without complete analyzable multi-shell dMRI data in age, sex, MoCA score, severity of cognitive impairment, or the assessed vascular risk factors (all *p* > 0.05; Supplementary Table S1).

The control group consisted of 36 healthy volunteers matched for age and sex, for whom a corresponding set of diffusion MRI indices was available.

### 3.2. Comparison of Diffusion Metric Values Along Three Corpus Callosum Segments for the SVD MRI Types and Controls

Given the differences between MRI types in age and sex, a separate general linear model adjusted for these factors was constructed for each dMRI metric. The primary analysis included 48 global dMRI values, corresponding to 16 diffusion metrics across three segments of the corpus callosum.

The age- and sex-adjusted overall group effect, comparing MRI Type 1, MRI Type 2, and controls, remained statistically significant after Benjamini–Hochberg correction for 45 of the 48 global dMRI values. For the corresponding models, pairwise between-group comparisons were performed with Bonferroni correction. The direction of statistically significant differences among all three groups is presented in Table 2; complete results, including adjusted difference values, 95% confidence intervals, and *p*-values, are presented in Supplementary Table S2.

For most values, changes in both MRI types were unidirectional relative to controls, but were more pronounced in MRI Type 1. Specifically, FA, INTRA, MK, RK, and AWF values were decreased in both MRI types compared with controls and were further decreased in MRI Type 1 relative to MRI Type 2. Conversely, MD, RD, EXTRA_MD, and EXTRA_TRANS were increased in both MRI types compared with controls and showed even higher values in MRI Type 1. A similar profile was observed for NDI in the forceps major and body, and for radEAD in the forceps minor and body. For certain metrics, including AD, AK, ODI, ISO, and axEAD, differences relative to controls or between MRI types depended on the corpus callosum segment and were less consistent. Of the 45 values with a significant overall group effect, 36 retained statistically significant differences between MRI Type 1 and MRI Type 2 after Bonferroni correction. For all 36 values that remained statistically significant, the overall group effect size was large (partial η^2^ ≥ 0.14). The ten measures with the highest partial η^2^ values are presented in Table 3; complete results are presented in Supplementary Table S2.

Notably, the most pronounced differences were predominantly localized in the middle segment of the corpus callosum. In MRI Type 1. the body of the corpus callosum showed lower values of AWF. INTRA, MK, FA, and RK, and higher values of EXTRA_TRANS, RD, MD, and radEAD compared with MRI Type 2; additionally, a lower AWF value was found in the forceps major.

To visualize the spatial distribution of these differences along the tract, node-wise profiles of dMRI metrics were constructed over the 5–95% segment of tract length. Each tract was represented by 100 nodes, allowing visualization of the variation in metric values along the corpus callosum.

**Figure 2:**
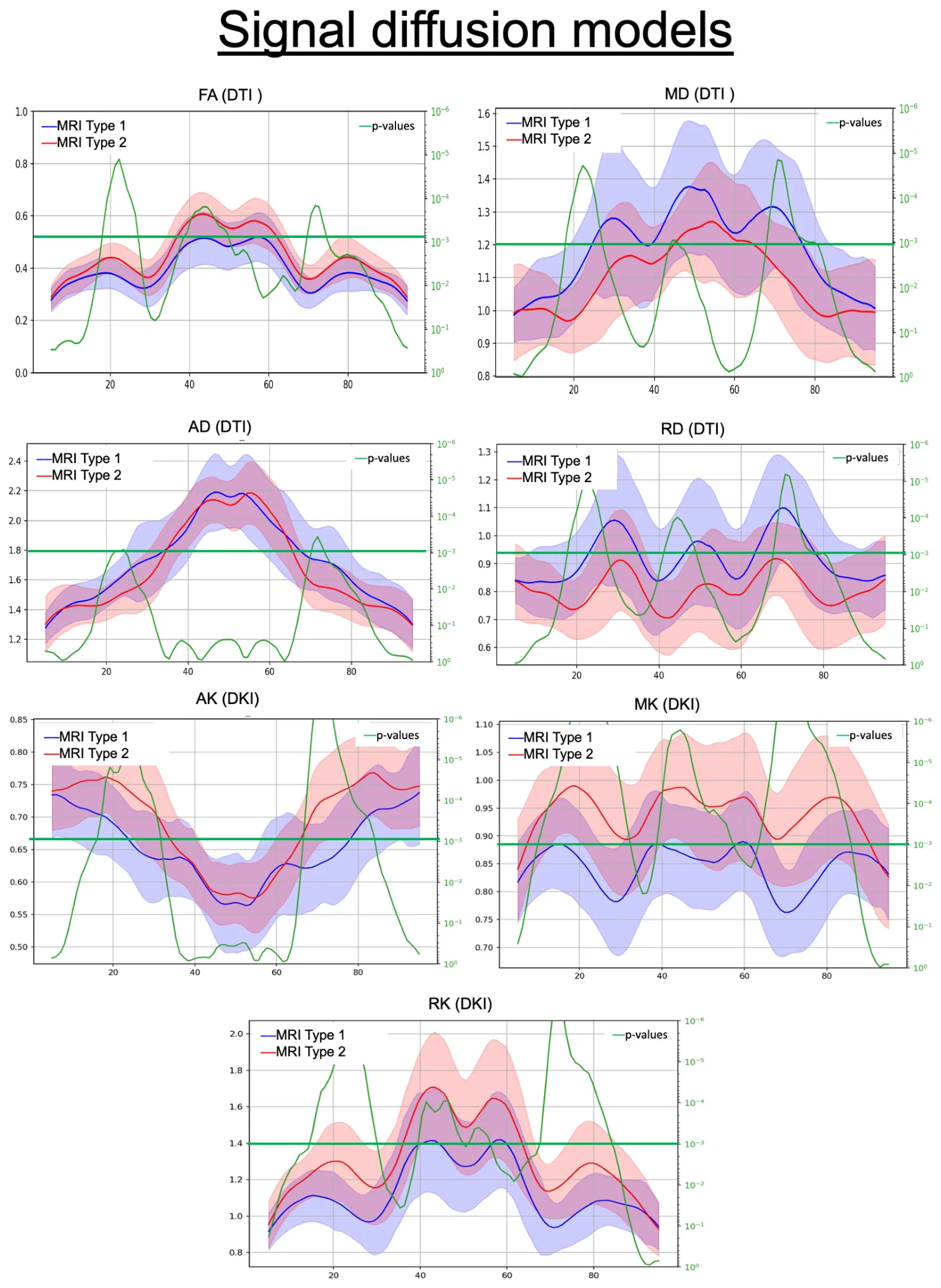
Spatial profiles of signal diffusion model metrics (DTI and DKI) along the body of the corpus callosum in patients with MRI Type 1 and MRI Type 2. Profiles are shown over the 5–95% segment of tract length. Values above the green horizontal line indicate nominally significant node-wise differences (*p* < 0.05).

**Figure 3:**
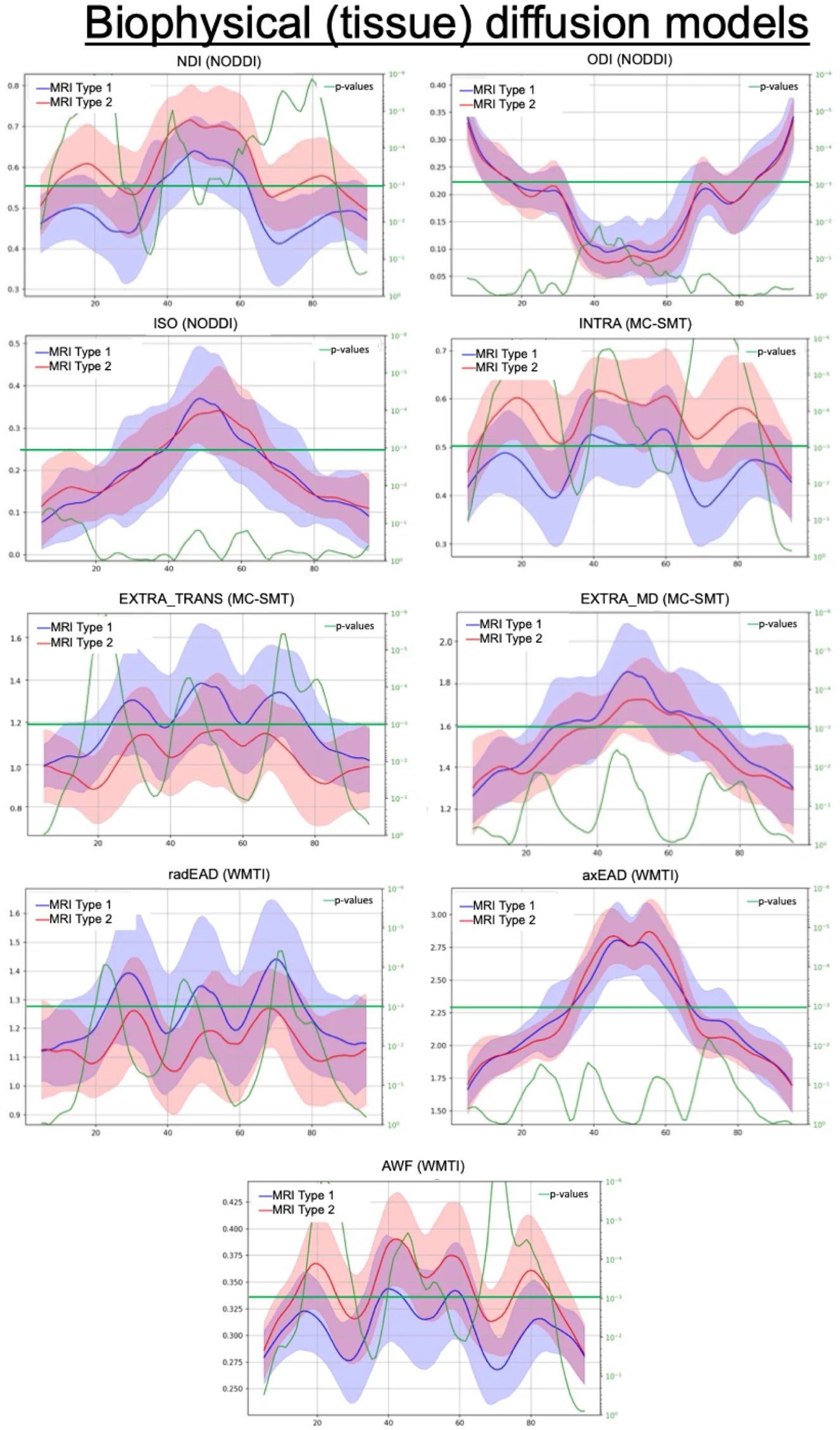
Spatial profiles of biophysical diffusion model metrics (NODDI, MC-SMT, and WMTI) along the body of the corpus callosum in patients with MRI Type 1 and MRI Type 2. Profiles are shown over the 5–95% segment of tract length. Values above the green horizontal line indicate nominally significant node-wise differences (*p* < 0.05).

The profiles for the body of the corpus callosum, where the most pronounced global differences were observed, are shown in Figure 2 and Figure 3.

The node-wise profiles were used for descriptive visualization. Point-wise comparisons between MRI Type 1 and MRI Type 2 were not used for confirmatory statistical inference, as they did not account for the age and sex imbalance between the MRI types, correction for multiple comparisons, or the spatial dependence between adjacent nodes. Therefore, formal spatially resolved statistical inference was based on three non-overlapping tract intervals (20–40%, 40–60%, and 60–80%), analyzed using age- and sex-adjusted general linear models with correction for multiple comparisons. After Benjamini–Hochberg correction, the overall group effect remained significant for 131 of 144 segmental values. In subsequent pairwise comparisons, 108 of these showed statistically significant differences between MRI Type 1 and MRI Type 2 after Bonferroni correction; for 103 of these 108 values, the overall group effect size was large (partial η^2^ ≥ 0.14). The results of the adjusted interval-based analysis were therefore generally consistent with the spatial patterns observed in the descriptive node-wise tract profiles. Complete results are presented in Supplementary Table S3, while the ten measures with the highest partial η^2^ values are presented in Table 4.

All 10 segmental measures with the highest partial η^2^ values were localized in the middle part of the corpus callosum, within the 60–80% segment of tract length. The segmental analysis generally replicated the findings of the global analysis: all nine body-of-corpus-callosum measures that were part of the global top 10 again showed the largest effects within the 60–80% segment, and NDI additionally appeared among the ten most pronounced values. The direction of differences was also consistent: in MRI Type 1, AWF, MK, INTRA, RK, FA, and NDI values were lower, whereas EXTRA_TRANS, RD, radEAD, and MD were higher, compared with MRI Type 2. When compared with the control group, the segmental values also showed changes in the same direction as the global measures, with deviations predominantly more pronounced in MRI Type 1. A summary of the MRI Type 1 versus MRI Type 2 comparisons across the global and interval-based analyses is provided in Supplementary Table S5.

To assess the combined contribution of the global dMRI values to discrimination between MRI Type 1 and MRI Type 2, a regularized Elastic Net logistic regression model was constructed including 48 dMRI indices, age, and sex. The discriminative performance of the model was assessed using repeated nested cross-validation.

**Figure 4:**
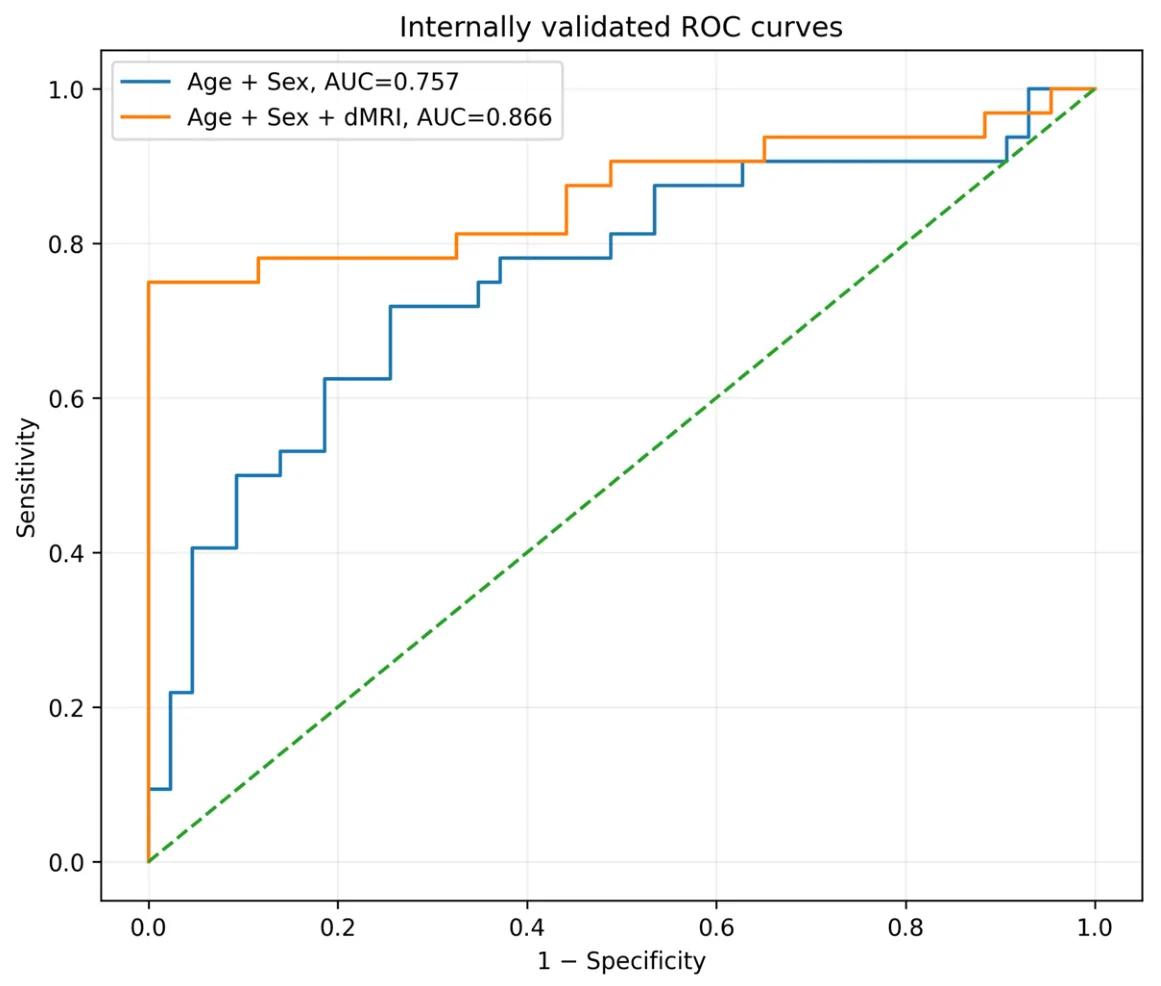
ROC curves of internally validated models for discriminating between MRI Type 1 and MRI Type 2.

The model including age, sex, and dMRI measures had an AUC of 0.866 (95% CI 0.762–0.953), an overall accuracy of 86.7% (95% CI 78.7–93.3), a sensitivity of 75.0% (95% CI 60.0–88.9), and a specificity of 95.3% (95% CI 88.2–100.0). For the model including only age and sex, the corresponding values were an AUC of 0.757 (95% CI 0.637–0.873), an overall accuracy of 73.3% (95% CI 62.7–82.7), a sensitivity of 71.9% (95% CI 56.0–87.5), and a specificity of 74.4% (95% CI 59.5–87.2). Thus, the inclusion of dMRI measures was associated with an increase in AUC of 0.109 and in overall accuracy of 13.4 percentage points (Figure 4).

Calibration analysis showed a Brier score of 0.135, a calibration intercept of 0.096, and a calibration slope of 1.513 for the model including age, sex, and dMRI measures. For the age- and sex-only model, the corresponding values were 0.199, −0.032, and 0.844, respectively.

Across repeated Elastic Net model construction, a set of interrelated dMRI features was consistently retained (Supplementary Table S4). Ten values had a selection frequency of ≥70%: NDI in the body, MK in the forceps major, INTRA in the body and forceps major, RK in the forceps major, radEAD, RD, EXTRA_TRANS and FA in the forceps minor, and AWF in the forceps major (Table 5).

Age was retained in 88% of outer CV iterations, whereas sex was retained in 12%. The complete feature selection frequencies are presented in Supplementary Table S4. Thus, the statistically significant differences we found between MRI Types 1 and 2 in the signal and biophysical diffusion model parameters further characterize the distinctions between them (Supplementary Table S6).

## 4. Discussion

The present study continues our series of investigations aimed at identifying ways to differentiate between different forms of age-related SVD. Their rationale stems from the pronounced clinical heterogeneity of patients with similar MRI changes on visual assessment. In this work, the dMRI method confirmed that the SVD MRI types at Fazekas stage 3 previously identified by us through hierarchical analysis of MRI features assessed by severity and localization [20] also differ in the severity of microstructural white matter damage, as demonstrated using the largest tract—the corpus callosum. Importantly, the identified differences persisted after statistical adjustment for baseline differences in age and sex and correction for multiple comparisons. Compared with MRI Type 2, MRI Type 1 was characterized by greater nerve fiber damage and, accordingly, lower FA, NDI, INTRA, AWF, MK, AK, and RK values, and higher MD and RD values, as well as greater extracellular diffusion with increased EXTRA_TRANS. It should be noted that fiber damage was accompanied by changes in metrics reflecting both overall microstructural damage and those associated predominantly with myelin damage (MD, RD, EXTRA_TRANS) and axonal damage (NDI, INTRA, AWF, MK, AK, RK) [36].

These findings suggest that NDI, MK, INTRA, RK, radEAD, and AWF are the most promising metrics for differentiating MRI types; RD, EXTRA_TRANS, and FA also demonstrated some informative value. At the same time, AD, ISO, and ODI, despite showing differences between individual groups, were less robust, which currently precludes considering them as independent differentiation markers. Further evaluation of these metrics requires confirmation in a larger, independent sample.

The correspondence between these metrics and the predominance of changes in specific fiber components was previously established in experimental studies [37], which subsequently allowed changes in RD and AD to be regarded as parameters associated with disrupted myelin organization and axonal damage, respectively [38,39]; however, their interpretation as direct morphological equivalents require caution. The identified changes were observed in all three segments of the corpus callosum, with more pronounced differences in the body of the corpus callosum, which may indicate a widespread nature of the microstructural damage.

Comparison of microstructural changes between the two forms of SVD relative to controls revealed a unidirectional pattern of pathological changes, with these changes being more pronounced in MRI Type 1. MRI Type 1 was characterized by a greater decrease in INTRA and a greater increase in EXTRA_TRANS, which may correspond to greater damage to the intra-axonal and extra-axonal compartments, respectively. At the same time, these metrics are model-dependent indirect characteristics of tissue microstructure, so their interpretation as direct morphological equivalents also require caution. The more pronounced changes in MRI Type 1 were not explained by age differences alone: despite the younger age of patients in this group, between-group differences persisted after including age and sex in the general linear models. Patients with both forms had WMH of Fazekas stage 3 and did not differ statistically significantly in the severity of vascular risk factors.

The greater severity of microstructural damage in MRI Type 1 is also consistent with a more severe clinical phenotype. Thus, dementia was diagnosed in nearly half of the patients with MRI Type 1. The identified unidirectional profile of microstructural changes in SVD relative to controls, together with the differences between its two forms, can be considered, on the one hand, as reflecting a pathophysiological continuum of this condition, and on the other hand, as corresponding to differences in the predominance, across MRI Types, of the previously established main mechanisms of brain damage—ischemia (hypoxia) due to arteriolosclerosis and endothelial dysfunction with sterile vascular inflammation and subsequent BBB damage and neuroinflammation. It can be hypothesized that the more pronounced microstructural changes in MRI Type 1 are related to a greater contribution of small vessel remodeling with chronic ischemia/hypoxia to its formation, whereas in MRI Type 2, mechanisms of BBB damage and neuroinflammation predominate. Indirect support for this reasoning may be provided by our previously obtained data on the association of MRI Type 1 with decreased VEGF-A, indicating severe vascular wall damage with endothelial loss, and of MRI Type 2 with TNF-α, a key inflammatory molecule involved in BBB damage [23], as well as with increased BBB permeability according to dynamic T1-weighted contrast-enhanced imaging [21,22]. This interpretation should be regarded as a pathophysiological hypothesis based on the totality of the present and previously obtained data, rather than as direct evidence of a specific mechanism of tissue damage.

At the same time, current understanding of SVD pathogenesis suggests the involvement of not only chronic ischemia, endothelial dysfunction, and neuroinflammation, but also glymphatic dysfunction, impaired interstitial clearance, and other processes [17,18,19]. These processes may potentially affect the distribution of intracellular and extracellular water and, accordingly, the diffusion characteristics of white matter. However, glymphatic function and the corresponding cellular and circulating biomarkers were not directly assessed in the present study, so their possible contribution is considered only as an additional pathophysiological hypothesis [17,18,19].

Both DTI and DKI parameters proved to be informative for distinguishing between the study groups. The latter are rarely used in clinical studies. The most consistent kurtosis parameter changes relative to controls were observed for MK and RK, whose values were decreased in both MRI types and to a greater extent in MRI Type 1; for AK, the pattern of differences was less uniform and depended on the corpus callosum segment. These findings are consistent with the results of several studies that also reported decreased MK and RK in areas of axonal loss in SVD patients with cognitive impairment [37,40,41]. Among the classical DTI metrics, the most pronounced differences between MRI types concerned FA, RD, and MD. Overall, RD and MD increased progressively, and FA decreased progressively, from controls to MRI Type 2 and further to MRI Type 1, across all three corpus callosum segments.

Our choice of the corpus callosum as the model tract was not incidental. On the one hand, it is a large tract with highly coherent fibers, which provides optimal conditions for constructing biophysical diffusion models; on the other hand, its microstructural characteristics have been shown to have the strongest association with cognitive impairment in SVD [26]. At the same time, restricting the analysis to the corpus callosum does not allow the identified patterns to be directly extrapolated to other projection and association tracts; the reproducibility of these changes in other white matter regions requires separate investigation.

In the future, a comprehensive assessment of conventional MRI features together with microstructural dMRI characteristics may expand the possibilities for phenotyping SVD patients and complement vascular and circulating biomarkers of the disease [17,18,19].

### Study Limitations

The present study has several limitations. First, its cross-sectional design does not allow assessment of the temporal sequence of microstructural changes or their prognostic significance. The dMRI analysis was performed in a relatively small sample, which may limit the generalizability of the findings. Although the multivariate model underwent internal validation, independent external validation was not performed. In addition, the analysis was restricted to the corpus callosum; therefore, the observed patterns cannot be fully extrapolated to other white matter tracts. Furthermore, node-wise tract profiles were used for descriptive visualization only and were not used for inferential statistical conclusions, because point-wise comparisons did not account for age and sex differences between the MRI types, multiple testing, or spatial dependence between adjacent nodes. Inferential conclusions regarding the spatial distribution of differences were therefore based on the age- and sex-adjusted interval-level analysis with correction for multiple comparisons. dMRI metrics are indirect markers of tissue microstructure and lack direct histopathological validation in the present study. The values of the biophysical parameters may potentially have been affected by partial-volume effects, increased free water, and violations of model assumptions in the presence of severe tissue damage and atrophy. Finally, cerebral perfusion, blood–brain barrier permeability, glymphatic function, inflammatory markers, and circulating vascular biomarkers were not directly assessed in the present study; therefore, the proposed pathophysiological interpretation requires confirmation in longitudinal multimodal studies.

## 5. Conclusions

Thus, the use of signal- and diffusion-based dMRI models to compare the two forms (MRI types) of SVD and the control group demonstrated a unidirectional pattern of pathological changes consistent with impaired microstructural organization of the white matter, with greater alterations observed in MRI Type 1. The identified differences persisted after adjustment for age and sex and correction for multiple comparisons. Multivariate analysis showed that differentiation between MRI types was determined by the combined contribution of interrelated dMRI metrics rather than by one or two isolated markers. Further studies are needed to elucidate the pathogenetic basis underlying the development of the two forms (MRI types) of SVD, which, despite the absence of differences in the severity of vascular risk factors, are associated with distinct diagnostic MRI patterns and differences in disease severity. The prognostic significance of the identified MRI phenotypes requires further investigation. These findings support further refinement of the diagnostic criteria for MRI phenotypes and assessment of the reproducibility of the identified microstructural differences in independent cohorts, with the ultimate goal of developing pathogenetically informed strategies for prevention and treatment.

## Figures and Tables

**Table 1:** Clinical characteristics of the participants included in the diffusion MRI analysis.

Parameter	MRI Type 1 (*n* = 43)	MRI Type 2 (*n* = 32)	*p*
Age (years ± SD)	60.7 ± 6.1	66.2 ± 6.0	<0.001
Men (*n*, %)	28 (65.1%)	13 (40.6%)	0.002
MoCA score (Me, [Q1; Q3])	21 [17; 25]	25 [22.75; 27.0]	<0.001
Severity of CI (*n*, %)			<0.001
SubCI	7 (16.3%)	13 (40.6%)	
MCI	13 (30.2%)	16 (50.0%)	
Dementia	23 (53.5%)	3 (9.4%)	
Arterial hypertension (*n*, %)	43 (100.0%)	31 (96.9%)	0.244
Type 2 diabetes mellitus (*n*, %)	8 (18.6%)	9 (28.1%)	0.331
Hypercholesterolemia (*n*, %)	28 (65.1%)	21 (65.6%)	0.964
Smoking (*n*, %)	12 (27.9%)	8 (25.0%)	0.779
Obesity (*n*, %)	13 (30.2%)	15 (46.9%)	0.141

Note. CI—cognitive impairment; MCI—mild cognitive impairment; SubCI—subjective cognitive impairment; MoCA—Montreal Cognitive Assessment; SD—standard deviation; Me—median; Q1—first quartile; Q3—third quartile.

**Table 2:** Direction of adjusted differences in global dMRI values between MRI Type 1, MRI Type 2, and controls.

Method	Metrics	Forceps Major	Forceps Minor	Body
DTI	FA	MRI Type 1 < MRI Type 2 < Control	MRI Type 1 < MRI Type 2 < Control	MRI Type 1 < MRI Type 2 < Control
DTI	AD	MRI Type 1 > Control	MRI Type 1 > MRI Type 2; MRI Type 1 > Control	Control < MRI Type 2 < MRI Type 1
DTI	MD	Control < MRI Type 2 < MRI Type 1	Control < MRI Type 2 < MRI Type 1	Control < MRI Type 2 < MRI Type 1
DTI	RD	Control < MRI Type 2 < MRI Type 1	Control < MRI Type 2 < MRI Type 1	Control < MRI Type 2 < MRI Type 1
NODDI	NDI	MRI Type 1 < MRI Type 2 < Control	MRI Type 1 < Control	MRI Type 1 < MRI Type 2 < Control
NODDI	ODI	MRI Type 1 > Control	NS	MRI Type 1 > Control
NODDI	ISO	MRI Type 1 > Control	MRI Type 1 > Control	MRI Type 1 > Control; MRI Type 2 > Control
MC-SMT	INTRA	MRI Type 1 < MRI Type 2 < Control	MRI Type 1 < MRI Type 2 < Control	MRI Type 1 < MRI Type 2 < Control
MC-SMT	EXTRA_MD	Control < MRI Type 2 < MRI Type 1	Control < MRI Type 2 < MRI Type 1	Control < MRI Type 2 < MRI Type 1
MC-SMT	EXTRA_TRANS	Control < MRI Type 2 < MRI Type 1	Control < MRI Type 2 < MRI Type 1	Control < MRI Type 2 < MRI Type 1
DKI	MK	MRI Type 1 < MRI Type 2 < Control	MRI Type 1 < MRI Type 2 < Control	MRI Type 1 < MRI Type 2 < Control
DKI	AK	MRI Type 1 < MRI Type 2; MRI Type 1 < Control	MRI Type 1 < MRI Type 2; MRI Type 1 < Control	MRI Type 1 < MRI Type 2 < Control
DKI	RK	MRI Type 1 < MRI Type 2 < Control	MRI Type 1 < MRI Type 2 < Control	MRI Type 1 < MRI Type 2 < Control
WMTI	AWF	MRI Type 1 < MRI Type 2 < Control	MRI Type 1 < MRI Type 2 < Control	MRI Type 1 < MRI Type 2 < Control
WMTI	axEAD	NS	NS	MRI Type 1 > Control; MRI Type 2 > Control
WMTI	radEAD	NS	Control < MRI Type 2 < MRI Type 1	Control < MRI Type 2 < MRI Type 1

Note. The “<“ and “>” signs indicate the direction of statistically significant pairwise differences after Bonferroni correction only. When all three pairwise comparisons were statistically significant, the groups are presented as a single sequence (e.g., MRI Type 1 < MRI Type 2 < Control). When not all pairwise comparisons were significant, only the confirmed comparisons are shown, separated by “;”. NS indicates that no statistically significant pairwise differences were detected or that the overall group effect did not remain significant after FDR correction. All models were adjusted for age and sex.

**Table 3:** Ten global dMRI measures with the highest partial η^2^ values.

Metrics	CC Part	F	Partial η^2^	MRI Type 1 − MRI Type 2 [95% CI]
EXTRA_TRANS	Body	88.255	0.645	0.172 [0.109; 0.236]
AWF	Body	87.567	0.644	−0.040 [−0.053; −0.027]
INTRA	Body	86.459	0.641	−0.094 [−0.123; −0.066]
MK	Body	86.318	0.640	−0.096 [−0.127; −0.065]
FA	Body	78.524	0.618	−0.060 [−0.080; −0.039]
RD	Body	67.805	0.598	0.133 [0.084; 0.182]
MD	Body	67.608	0.582	0.107 [0.064; 0.149]
radEAD	Body	67.389	0.581	0.128 [0.076; 0.179]
AWF	Forceps major	67.710	0.563	−0.048 [−0.064; −0.031]
RK	Body	61.977	0.561	−0.191 [−0.263; −0.118]

Note. For all measures presented in the table, FDR q < 0.001 and the Bonferroni-adjusted *p*-value for the comparison between MRI Type 1 and MRI Type 2 was <0.001. Diffusivity metrics MD, RD, radEAD, EXTRA_TRANS were expressed in ×10^−3^ mm^2^/s,; the remaining metrics were dimensionless.

**Table 4:** Ten segmental dMRI measures with the highest partial η^2^ values.

Metrics	CC Part	Interval	F	Partial η^2^	MRI Type 1 − MRI Type 2 [95% CI]
AWF	Body	60–80%	117.691	0.708	−0.046 [−0.060; −0.031]
MK	Body	60–80%	116.847	0.707	−0.119 [−0.155; −0.082]
INTRA	Body	60–80%	106.958	0.688	−0.119 [−0.155; −0.083]
EXTRA_TRANS	Body	60–80%	103.683	0.681	0.228 [0.150; 0.306]
RK	Body	60–80%	90.868	0.652	−0.193 [−0.270; −0.116]
FA	Body	60–80%	90.204	0.650	−0.063 [−0.086; −0.039]
RD	Body	60–80%	82.061	0.643	0.184 [0.124; 0.244]
radEAD	Body	60–80%	84.328	0.635	0.181 [0.120; 0.242]
NDI	Body	60–80%	79.737	0.622	−0.092 [−0.127; −0.057]
MD	Body	60–80%	76.717	0.613	0.167 [0.111; 0.222]

Note. For all measures presented in the table, FDR q < 0.001 and the Bonferroni-adjusted *p*-value for the comparison between MRI Type 1 and MRI Type 2 was <0.001; CC—corpus callosum. Diffusivity metrics MD, RD, radEAD, EXTRA_TRANS were expressed in ×10^−3^ mm^2^/s; the remaining metrics were dimensionless.

**Table 5:** Stability of feature selection in the primary Elastic Net model.

Feature	Number of Outer CV Iterations (Out of 50)	Selection Frequency
NDI_CC_Body_5_95	50	100.0%
MK_CC_ForcepsMajor_5_95	49	98.0%
INTRA_CC_Body_5_95	45	90.0%
INTRA_CC_ForcepsMajor_5_95	44	88.0%
RK_CC_ForcepsMajor_5_95	44	88.0%
radEAD_CC_ForcepsMinor_5_95	42	84.0%
AWF_CC_ForcepsMajor_5_95	41	82.0%
RD_CC_ForcepsMinor_5_95	38	76.0%
EXTRA_TRANS_CC_ForcepsMinor_5_95	37	74.0%
FA_CC_ForcepsMinor_5_95	35	70.0%

## Data Availability

All data relevant to this study are included in the article. Additional information may be obtained from the corresponding author upon reasonable request, subject to patient confidentiality and applicable data protection regulations.

## Supplementary Materials

The following supporting information can be downloaded at: https://www.mdpi.com/article/10.3390/diagnostics16172861/s1, Table S1. Comparison of baseline clinical and demographic characteristics between patients included and not included in the dMRI analysis; Table S2. Complete results of the GLM and pairwise comparisons for 48 global 5–95% measures; Table S3. Complete results of the GLM and pairwise comparisons for 144 segmental metrics; Table S4. Stability of feature selection in the primary Elastic Net model; Table S5. Summary comparison of MRI Type 1 and MRI Type 2 for models retaining an overall effect after FDR correction; Table S6. Generalized characteristics of SVD MRI types at Fazekas stage 3, based on the results of the present study and earlier studies by the author group [20,21,22,23].

## Abbreviations

The following abbreviations are used in this manuscript:

SVD	Cerebral small vessel disease
dMRI	Diffusion MRI
DTI	Diffusion Tensor Imaging
DKI	Diffusion Kurtosis Imaging
NODDI	Neurite Orientation Dispersion and Density Imaging
WMTI	White Matter Tract Integrity
MC-SMT	Multi-compartment Spherical Mean Technique
FA	Fractional anisotropy
NDI	Neurite density index
INTRA	Intra-axonal volume fraction
AWF	Axonal water fraction
MK	Mean kurtosis
AK	Axial kurtosis
RK	Radial kurtosis
MD	Mean diffusivity
RD	Radial diffusivity
EXTRA_TRANS	Extra-axonal transverse diffusivity
EXTRA_MD	Extra-axonal mean diffusivity
WMH	White matter hyperintensity
radEAD	extra-axonal radial diffusivity
axEAD	extra-axonal axial diffusivity
ODI	Orientation dispersion index
ISO	Cerebrospinal fluid fraction
AUC	area under the curve
NAWM	normal-appearing white matter
Ktrans	capillary permeability coefficient
CC	Corpus callosum
CI	Cognitive impairment
BBB	Blood-brain barrier permeability
VEGF-A	Vascular endothelial growth factor-A
TNF-α	tumor necrosis factor-alpha
