# Supplementary material for: Microstructural Changes in the Corpus Callosum in Different Forms of Sporadic Age-Related Cerebral Small Vessel Disease
Source: Diagnostics (Basel). 2026 Sep 5;16(17):2861. doi: 10.3390/diagnostics16172861 (PMC13565003; doi:10.3390/diagnostics16172861)
Supplement: Supplementary file 1 [file diagnostics-16-02861-s001.zip › diagnostics-4471399-supplementary.pdf]

Supplementary

Table S1. Comparison of baseline clinical and demographic characteristics between patients included and not included in the dMRI analysis

Parameter	Included in dMRI analysis (n=75)	Not included in dMRI analysis (n=39)	p
Age (years ± SD)	62.9±6.5	60.3±7.8	0.095
Men (n, %)	41 (54.7%)	23 (59.0%)	0.660
MoCA score (Me, [Q1; Q3])	23 [20; 26]	23 [19; 26]	0.742
Severity of CI, (n, %)			0.288
— SubCI	20 (26.7%)	12 (30.8%)	
— MCI	29 (38.7%)	19 (48.7%)	
— Dementia	26 (34.7%)	8 (20.5%)	
Arterial hypertension, (n, %)	74 (98.7%)	37 (94.9%)	0.269
Type 2 diabetes mellitus, (n, %)	17 (22.7%)	7 (17.9%)	0.558
Hypercholesterolemia, (n, %)	49 (65.3%)	25 (64.1%)	0.896
Smoking, (n, %)	20 (26.7%)	5 (12.8%)	0.090
Obesity, (n, %)	28 (37.3%)	19 (48.7%)	0.241

Note. CI – cognitive impairment; MCI – mild cognitive impairment; SubCI – subjective cognitive impairment; MoCA – Montreal Cognitive Assessment; SD – standard deviation; Me – median; Q1 – first quartile; Q3 – third quartile.

Table S2. Complete results of the GLM and pairwise comparisons for 48 global 5–95% measures

Metrics	CC part	F	partial η^2	p	FDR q	MRI Type 1 vs MRI Type 2, difference [95% CI]	p Bonf.	MRI Type 1 vs Control, difference [95% CI]	p Bonf.	MRI Type 2 vs Control, difference [95% CI]	p Bonf.
FA	Forceps major	49.417	0.485	<0.001	<0.001	-0.059 [-0.087; -0.032]	<0.001	-0.130 [-0.156; -0.104]	<0.001	-0.071 [-0.100; -0.041]	<0.001
FA	Forceps minor	52.194	0.499	<0.001	<0.001	-0.043 [-0.065; -0.022]	<0.001	-0.104 [-0.124; -0.084]	<0.001	-0.061 [-0.084; -0.037]	<0.001
FA	Body	78.524	0.618	<0.001	<0.001	-0.060 [-0.080; -0.039]	<0.001	-0.123 [-0.142; -0.103]	<0.001	-0.063 [-0.085; -0.041]	<0.001
AD	Forceps major	8.806	0.144	<0.001	<0.001	0.081 [0.006; 0.156]	0.103	0.150 [0.078; 0.222]	<0.001	0.069 [-0.013; 0.151]	0.298
AD	Forceps minor	20.010	0.276	<0.001	<0.001	0.082 [0.035; 0.128]	0.002	0.137 [0.093; 0.181]	<0.001	0.056 [0.005; 0.106]	0.098
AD	Body	34.217	0.414	<0.001	<0.001	0.057 [0.017; 0.097]	0.018	0.155 [0.117; 0.192]	<0.001	0.098 [0.056; 0.140]	<0.001

MD	Forceps major	26.933	0.339	<0.001	<0.001	0.121 [0.052; 0.190]	0.002	0.241 [0.176; 0.307]	<0.001	0.120 [0.045; 0.195]	0.006
MD	Forceps minor	45.597	0.465	<0.001	<0.001	0.103 [0.059; 0.148]	<0.001	0.200 [0.158; 0.241]	<0.001	0.096 [0.048; 0.145]	<0.001
MD	Body	67.608	0.582	<0.001	<0.001	0.107 [0.064; 0.149]	<0.001	0.231 [0.192; 0.270]	<0.001	0.124 [0.080; 0.169]	<0.001
RD	Forceps major	30.893	0.384	<0.001	<0.001	0.141 [0.066; 0.216]	<0.001	0.287 [0.214; 0.360]	<0.001	0.146 [0.063; 0.229]	0.002
RD	Forceps minor	45.982	0.482	<0.001	<0.001	0.115 [0.065; 0.165]	<0.001	0.232 [0.184; 0.280]	<0.001	0.117 [0.062; 0.173]	<0.001
RD	Body	67.805	0.598	<0.001	<0.001	0.133 [0.084; 0.182]	<0.001	0.274 [0.227; 0.321]	<0.001	0.141 [0.089; 0.192]	<0.001
NDI	Forceps major	28.135	0.349	<0.001	<0.001	-0.069 [-0.102; -0.036]	<0.001	-0.117 [-0.148; -0.085]	<0.001	-0.048 [-0.084; -0.012]	0.030
NDI	Forceps minor	10.257	0.163	<0.001	<0.001	-0.057 [-0.110; -0.005]	0.095	-0.112 [-0.161; -0.063]	<0.001	-0.054 [-0.112; 0.003]	0.184
NDI	Body	61.444	0.559	<0.001	<0.001	-0.089 [-0.119; -0.059]	<0.001	-0.154 [-0.182; -0.126]	<0.001	-0.065 [-0.096; -0.034]	<0.001
ODI	Forceps major	4.587	0.080	0.012	0.013	0.010 [-0.002; 0.023]	0.276	0.017 [0.006; 0.029]	0.011	0.007 [-0.006; 0.020]	0.912
ODI	Forceps minor	2.304	0.042	0.104	0.110	0.003 [-0.017; 0.024]	1.000	0.021 [0.001; 0.040]	0.117	0.017 [-0.005; 0.040]	0.402
ODI	Body	5.668	0.105	0.005	0.005	0.010 [-0.002; 0.021]	0.306	0.018 [0.007; 0.029]	0.003	0.009 [-0.003; 0.020]	0.475
ISO	Forceps major	9.015	0.147	<0.001	<0.001	0.028 [-0.009; 0.065]	0.408	0.075 [0.040; 0.110]	<0.001	0.047 [0.007; 0.088]	0.064
ISO	Forceps minor	5.225	0.091	0.007	0.008	0.023 [-0.011; 0.057]	0.570	0.053 [0.020; 0.085]	0.005	0.030 [-0.008; 0.067]	0.348
ISO	Body	8.250	0.145	<0.001	<0.001	0.006 [-0.021; 0.033]	1.000	0.049 [0.024; 0.074]	<0.001	0.043 [0.015; 0.071]	0.010
INTRA	Forceps major	63.084	0.546	<0.001	<0.001	-0.099 [-0.132; -0.066]	<0.001	-0.175 [-0.207; -0.144]	<0.001	-0.076 [-0.112; -0.040]	<0.001
INTRA	Forceps minor	31.077	0.372	<0.001	<0.001	-0.076 [-0.117; -0.036]	<0.001	-0.151 [-0.190; -0.113]	<0.001	-0.075 [-0.119; -0.031]	0.003

INTRA	Body	86.459	0.641	<0.001	<0.001	-0.094 [-0.123; -0.066]	<0.001	-0.176 [-0.203; -0.149]	<0.001	-0.081 [-0.111; -0.052]	<0.001
EXTRA_MD	Forceps major	31.762	0.377	<0.001	<0.001	0.114 [0.044; 0.183]	0.005	0.268 [0.201; 0.334]	<0.001	0.154 [0.078; 0.231]	<0.001
EXTRA_MD	Forceps minor	40.231	0.434	<0.001	<0.001	0.097 [0.045; 0.149]	0.001	0.223 [0.174; 0.273]	<0.001	0.126 [0.069; 0.183]	<0.001
EXTRA_MD	Body	53.441	0.524	<0.001	<0.001	0.075 [0.024; 0.125]	0.014	0.245 [0.197; 0.292]	<0.001	0.170 [0.117; 0.224]	<0.001
EXTRA_TRANS	Forceps major	49.434	0.485	<0.001	<0.001	0.207 [0.121; 0.294]	<0.001	0.412 [0.329; 0.494]	<0.001	0.204 [0.110; 0.299]	<0.001
EXTRA_TRANS	Forceps minor	60.462	0.535	<0.001	<0.001	0.170 [0.103; 0.237]	<0.001	0.348 [0.285; 0.411]	<0.001	0.178 [0.105; 0.251]	<0.001
EXTRA_TRANS	Body	88.255	0.645	<0.001	<0.001	0.172 [0.109; 0.236]	<0.001	0.397 [0.337; 0.456]	<0.001	0.224 [0.158; 0.291]	<0.001
MK	Forceps major	59.253	0.530	<0.001	<0.001	-0.128 [-0.169; -0.087]	<0.001	-0.209 [-0.248; -0.170]	<0.001	-0.081 [-0.126; -0.037]	0.001
MK	Forceps minor	30.474	0.367	<0.001	<0.001	-0.079 [-0.124; -0.033]	0.003	-0.169 [-0.212; -0.126]	<0.001	-0.090 [-0.140; -0.041]	0.002
MK	Body	86.318	0.640	<0.001	<0.001	-0.096 [-0.127; -0.065]	<0.001	-0.191 [-0.219; -0.162]	<0.001	-0.094 [-0.127; -0.062]	<0.001
AK	Forceps major	8.908	0.145	<0.001	<0.001	-0.032 [-0.055; -0.009]	0.023	-0.045 [-0.067; -0.023]	<0.001	-0.013 [-0.038; 0.012]	0.940
AK	Forceps minor	10.161	0.162	<0.001	<0.001	-0.039 [-0.069; -0.008]	0.042	-0.064 [-0.093; -0.035]	<0.001	-0.026 [-0.059; 0.008]	0.395
AK	Body	58.192	0.545	<0.001	<0.001	-0.037 [-0.054; -0.021]	<0.001	-0.084 [-0.099; -0.068]	<0.001	-0.046 [-0.063; -0.029]	<0.001
RK	Forceps major	53.750	0.506	<0.001	<0.001	-0.234 [-0.316; -0.152]	<0.001	-0.403 [-0.481; -0.324]	<0.001	-0.169 [-0.259; -0.079]	<0.001
RK	Forceps minor	48.166	0.478	<0.001	<0.001	-0.124 [-0.192; -0.055]	0.002	-0.321 [-0.385; -0.256]	<0.001	-0.197 [-0.272; -0.122]	<0.001
RK	Body	61.977	0.561	<0.001	<0.001	-0.191 [-0.263; -0.118]	<0.001	-0.377 [-0.445; -0.310]	<0.001	-0.187 [-0.262; -0.112]	<0.001

AWF	Forceps major	67.710	0.563	<0.001	<0.001	-0.048 [-0.064; -0.031]	<0.001	-0.093 [-0.109; -0.077]	<0.001	-0.045 [-0.063; -0.027]	<0.001
AWF	Forceps minor	65.443	0.555	<0.001	<0.001	-0.030 [-0.044; -0.017]	<0.001	-0.075 [-0.088; -0.062]	<0.001	-0.045 [-0.060; -0.030]	<0.001
AWF	Body	87.567	0.644	<0.001	<0.001	-0.040 [-0.053; -0.027]	<0.001	-0.081 [-0.094; -0.069]	<0.001	-0.041 [-0.055; -0.028]	<0.001
axEAD	Forceps major	0.400	0.009	0.672	0.672	-0.069 [-0.243; 0.105]	1.000	0.010 [-0.152; 0.172]	1.000	0.079 [-0.113; 0.271]	1.000
axEAD	Forceps minor	3.527	0.064	0.033	0.035	0.050 [0.004; 0.095]	0.100	0.049 [0.006; 0.093]	0.083	-0.0004 [-0.051; 0.050]	1.000
axEAD	Body	6.549	0.119	0.002	0.003	0.006 [-0.038; 0.050]	1.000	0.070 [0.029; 0.111]	0.003	0.064 [0.018; 0.109]	0.020
radEAD	Forceps major	0.459	0.009	0.633	0.647	0.038 [-0.182; 0.259]	1.000	0.096 [-0.103; 0.296]	1.000	0.058 [-0.180; 0.296]	1.000
radEAD	Forceps minor	31.171	0.373	<0.001	<0.001	0.116 [0.058; 0.174]	<0.001	0.215 [0.160; 0.270]	<0.001	0.099 [0.036; 0.162]	0.007
radEAD	Body	67.389	0.581	<0.001	<0.001	0.128 [0.076; 0.179]	<0.001	0.280 [0.232; 0.328]	<0.001	0.152 [0.099; 0.205]	<0.001

Note. FDR q refers to correction for multiple omnibus GLM comparisons; p Bonf. refers to pairwise post-hoc comparisons within the corresponding model. AD, MD, RD, axEAD, radEAD, EXTRA_MD and EXTRA_TRANS are expressed in ×10⁻³ mm²/s; . All other metrics are dimensionless.

Table S3. Complete results of the GLM and pairwise comparisons for 144 segmental metrics

Metrics	CC part	Interval	F	partial η²	p	FDR q	MRI Type 1 vs MRI Type 2, difference [95% CI]	p Bonf.	MRI Type 1 vs Control, difference [95% CI]	p Bonf.	MRI Type 2 vs Control, difference [95% CI]	p Bonf.
FA	Body	20–40%	56.752	0.539	<0.001	<0.001	-0.064 [-0.092; -0.037]	<0.001	-0.138 [-0.164; -0.113]	<0.001	-0.074 [-0.103; -0.045]	<0.001
FA	Body	40–60%	26.855	0.356	<0.001	<0.001	-0.094 [-0.132; -0.055]	<0.001	-0.128 [-0.165; -0.092]	<0.001	-0.035 [-0.075; 0.006]	0.270
FA	Body	60–80%	90.204	0.650	<0.001	<0.001	-0.063 [-0.086; -0.039]	<0.001	-0.150 [-0.172; -0.128]	<0.001	-0.087 [-0.112; -0.063]	<0.001
FA	Forceps major	20–40%	27.755	0.346	<0.001	<0.001	-0.065 [-0.105; -0.025]	0.005	-0.143 [-0.181; -0.105]	<0.001	-0.078 [-0.121; -0.034]	0.002

FA	Forceps major	40–60%	32.753	0.384	<0.001	<0.001	-0.086 [-0.127; -0.046]	<0.001	-0.155 [-0.194; -0.117]	<0.001	-0.069 [-0.113; -0.025]	0.007
FA	Forceps major	60–80%	36.167	0.408	<0.001	<0.001	-0.075 [-0.115; -0.035]	<0.001	-0.163 [-0.201; -0.125]	<0.001	-0.088 [-0.132; -0.044]	<0.001
FA	Forceps minor	20–40%	40.715	0.437	<0.001	<0.001	-0.053 [-0.082; -0.025]	<0.001	-0.122 [-0.149; -0.095]	<0.001	-0.068 [-0.100; -0.037]	<0.001
FA	Forceps minor	40–60%	33.831	0.392	<0.001	<0.001	-0.078 [-0.116; -0.039]	<0.001	-0.151 [-0.187; -0.114]	<0.001	-0.073 [-0.115; -0.031]	0.003
FA	Forceps minor	60–80%	59.180	0.530	<0.001	<0.001	-0.045 [-0.067; -0.023]	<0.001	-0.114 [-0.135; -0.093]	<0.001	-0.069 [-0.093; -0.045]	<0.001
AD	Body	20–40%	20.914	0.301	<0.001	<0.001	0.057 [-0.017; 0.132]	0.392	0.223 [0.153; 0.292]	<0.001	0.165 [0.088; 0.243]	<0.001
AD	Body	40–60%	0.013	0.0003	0.987	0.987	0.005 [-0.072; 0.082]	1.000	0.005 [-0.067; 0.077]	1.000	-0.0002 [-0.080; 0.080]	1.000
AD	Body	60–80%	35.192	0.420	<0.001	<0.001	0.136 [0.072; 0.200]	<0.001	0.252 [0.192; 0.312]	<0.001	0.116 [0.049; 0.183]	0.003
AD	Forceps major	20–40%	6.524	0.111	0.002	0.003	0.102 [-0.007; 0.211]	0.202	0.188 [0.084; 0.293]	0.002	0.086 [-0.033; 0.206]	0.467
AD	Forceps major	40–60%	13.548	0.205	<0.001	<0.001	0.202 [0.114; 0.290]	<0.001	0.172 [0.088; 0.257]	<0.001	-0.030 [-0.126; 0.067]	1.000
AD	Forceps major	60–80%	4.749	0.083	0.011	0.012	0.088 [-0.033; 0.208]	0.457	0.178 [0.063; 0.293]	0.008	0.091 [-0.041; 0.222]	0.529
AD	Forceps minor	20–40%	20.619	0.282	<0.001	<0.001	0.110 [0.052; 0.169]	<0.001	0.174 [0.118; 0.229]	<0.001	0.063 [-0.0006; 0.127]	0.156
AD	Forceps minor	40–60%	5.061	0.088	0.008	0.009	0.098 [0.023; 0.172]	0.034	0.096 [0.025; 0.167]	0.025	-0.002 [-0.084; 0.080]	1.000
AD	Forceps minor	60–80%	18.435	0.260	<0.001	<0.001	0.094 [0.032; 0.156]	0.010	0.177 [0.119; 0.235]	<0.001	0.083 [0.015; 0.150]	0.050
MD	Body	20–40%	38.388	0.442	<0.001	<0.001	0.115 [0.044; 0.186]	0.005	0.291 [0.225; 0.357]	<0.001	0.176 [0.102; 0.250]	<0.001
MD	Body	40–60%	14.096	0.225	<0.001	<0.001	0.115 [0.052; 0.177]	0.001	0.149 [0.091; 0.208]	<0.001	0.035 [-0.031; 0.100]	0.883
MD	Body	60–80%	76.717	0.613	<0.001	<0.001	0.167 [0.111; 0.222]	<0.001	0.325 [0.273; 0.377]	<0.001	0.158 [0.100; 0.216]	<0.001

MD	Forceps major	20–40%	16.084	0.235	<0.001	<0.001	0.146 [0.042; 0.251]	0.019	0.283 [0.183; 0.383]	<0.001	0.137 [0.022; 0.251]	0.058
MD	Forceps major	40–60%	38.708	0.424	<0.001	<0.001	0.218 [0.147; 0.289]	<0.001	0.277 [0.210; 0.345]	<0.001	0.059 [-0.018; 0.136]	0.395
MD	Forceps major	60–80%	17.282	0.248	<0.001	<0.001	0.144 [0.038; 0.251]	0.025	0.300 [0.199; 0.402]	<0.001	0.156 [0.040; 0.272]	0.027
MD	Forceps minor	20–40%	42.721	0.449	<0.001	<0.001	0.134 [0.078; 0.189]	<0.001	0.243 [0.190; 0.295]	<0.001	0.109 [0.048; 0.170]	0.002
MD	Forceps minor	40–60%	28.059	0.348	<0.001	<0.001	0.152 [0.085; 0.219]	<0.001	0.230 [0.167; 0.293]	<0.001	0.078 [0.005; 0.151]	0.109
MD	Forceps minor	60–80%	44.012	0.456	<0.001	<0.001	0.113 [0.061; 0.166]	<0.001	0.232 [0.183; 0.282]	<0.001	0.119 [0.062; 0.176]	<0.001
RD	Body	20–40%	40.919	0.473	<0.001	<0.001	0.146 [0.070; 0.223]	<0.001	0.332 [0.259; 0.405]	<0.001	0.186 [0.105; 0.266]	<0.001
RD	Body	40–60%	21.189	0.318	<0.001	<0.001	0.172 [0.095; 0.249]	<0.001	0.229 [0.155; 0.302]	<0.001	0.057 [-0.024; 0.138]	0.498
RD	Body	60–80%	82.061	0.643	<0.001	<0.001	0.184 [0.124; 0.244]	<0.001	0.367 [0.310; 0.424]	<0.001	0.183 [0.120; 0.246]	<0.001
RD	Forceps major	20–40%	18.038	0.267	<0.001	<0.001	0.170 [0.055; 0.284]	0.012	0.335 [0.223; 0.446]	<0.001	0.165 [0.039; 0.292]	0.033
RD	Forceps major	40–60%	35.122	0.415	<0.001	<0.001	0.226 [0.143; 0.309]	<0.001	0.327 [0.246; 0.409]	<0.001	0.101 [0.009; 0.194]	0.095
RD	Forceps major	60–80%	20.465	0.293	<0.001	<0.001	0.172 [0.059; 0.286]	0.010	0.355 [0.245; 0.466]	<0.001	0.183 [0.058; 0.309]	0.014
RD	Forceps minor	20–40%	41.686	0.457	<0.001	<0.001	0.146 [0.082; 0.209]	<0.001	0.279 [0.217; 0.340]	<0.001	0.133 [0.063; 0.203]	<0.001
RD	Forceps minor	40–60%	30.825	0.384	<0.001	<0.001	0.180 [0.099; 0.261]	<0.001	0.302 [0.224; 0.380]	<0.001	0.121 [0.032; 0.211]	0.024
RD	Forceps minor	60–80%	48.397	0.494	<0.001	<0.001	0.123 [0.069; 0.178]	<0.001	0.260 [0.207; 0.313]	<0.001	0.137 [0.077; 0.197]	<0.001
NDI	Body	20–40%	47.194	0.493	<0.001	<0.001	-0.109 [-0.150; -0.069]	<0.001	-0.181 [-0.218; -0.144]	<0.001	-0.072 [-0.113; -0.030]	0.003
NDI	Body	40–60%	20.721	0.299	<0.001	<0.001	-0.090 [-0.127; -0.053]	<0.001	-0.102 [-0.137; -0.068]	<0.001	-0.012 [-0.051; 0.026]	1.000

NDI	Body	60–80%	79.737	0.622	<0.001	<0.001	-0.092 [-0.127; -0.057]	<0.001	-0.208 [-0.240; -0.175]	<0.001	-0.115 [-0.152; -0.079]	<0.001
NDI	Forceps major	20–40%	21.500	0.291	<0.001	<0.001	-0.060 [-0.098; -0.022]	0.007	-0.119 [-0.156; -0.083]	<0.001	-0.059 [-0.101; -0.018]	0.017
NDI	Forceps major	40–60%	9.326	0.151	<0.001	<0.001	-0.077 [-0.129; -0.025]	0.012	-0.100 [-0.150; -0.051]	<0.001	-0.023 [-0.080; 0.033]	1.000
NDI	Forceps major	60–80%	33.326	0.388	<0.001	<0.001	-0.102 [-0.144; -0.059]	<0.001	-0.164 [-0.204; -0.123]	<0.001	-0.062 [-0.109; -0.015]	0.030
NDI	Forceps minor	20–40%	16.796	0.242	<0.001	<0.001	-0.073 [-0.128; -0.019]	0.027	-0.151 [-0.202; -0.099]	<0.001	-0.077 [-0.137; -0.017]	0.036
NDI	Forceps minor	40–60%	8.673	0.142	<0.001	<0.001	-0.066 [-0.123; -0.009]	0.070	-0.110 [-0.164; -0.057]	<0.001	-0.044 [-0.107; 0.018]	0.480
NDI	Forceps minor	60–80%	11.212	0.176	<0.001	<0.001	-0.071 [-0.128; -0.014]	0.045	-0.127 [-0.181; -0.073]	<0.001	-0.056 [-0.118; 0.007]	0.239
ODI	Body	20–40%	2.441	0.048	0.092	0.099	0.011 [-0.003; 0.024]	0.385	0.013 [0.0007; 0.026]	0.118	0.003 [-0.011; 0.017]	1.000
ODI	Body	40–60%	5.737	0.106	0.004	0.005	0.019 [0.004; 0.034]	0.038	0.022 [0.008; 0.035]	0.008	0.003 [-0.013; 0.018]	1.000
ODI	Body	60–80%	1.984	0.039	0.143	0.149	-0.0002 [-0.018; 0.018]	1.000	0.015 [-0.002; 0.032]	0.225	0.016 [-0.003; 0.034]	0.320
ODI	Forceps major	20–40%	2.033	0.037	0.136	0.143	0.009 [-0.007; 0.025]	0.748	0.015 [-0.0001; 0.030]	0.156	0.006 [-0.012; 0.023]	1.000
ODI	Forceps major	40–60%	3.003	0.054	0.054	0.058	0.005 [-0.009; 0.019]	1.000	0.016 [0.003; 0.029]	0.048	0.011 [-0.004; 0.026]	0.452
ODI	Forceps major	60–80%	2.876	0.052	0.061	0.065	0.013 [-0.007; 0.033]	0.634	0.023 [0.004; 0.043]	0.060	0.010 [-0.012; 0.033]	1.000
ODI	Forceps minor	20–40%	1.657	0.031	0.196	0.203	0.003 [-0.022; 0.028]	1.000	0.021 [-0.003; 0.045]	0.245	0.018 [-0.009; 0.046]	0.562
ODI	Forceps minor	40–60%	7.594	0.126	<0.001	0.001	0.018 [-0.003; 0.038]	0.256	0.038 [0.019; 0.057]	<0.001	0.020 [-0.002; 0.042]	0.240
ODI	Forceps minor	60–80%	2.116	0.039	0.126	0.133	0.002 [-0.022; 0.027]	1.000	0.023 [-0.00009; 0.047]	0.153	0.021 [-0.006; 0.048]	0.388

ISO	Body	20–40%	7.038	0.127	0.001	0.002	-0.0005 [-0.043; 0.042]	1.000	0.067 [0.028; 0.107]	0.003	0.068 [0.024; 0.112]	0.008
ISO	Body	40–60%	3.171	0.061	0.046	0.051	0.022 [-0.017; 0.062]	0.773	0.046 [0.010; 0.083]	0.040	0.024 [-0.017; 0.065]	0.745
ISO	Body	60–80%	11.503	0.192	<0.001	<0.001	0.031 [0.0007; 0.062]	0.135	0.069 [0.040; 0.097]	<0.001	0.038 [0.006; 0.069]	0.063
ISO	Forceps major	20–40%	6.847	0.115	0.002	0.002	0.047 [-0.007; 0.100]	0.258	0.095 [0.044; 0.146]	0.001	0.048 [-0.010; 0.107]	0.317
ISO	Forceps major	40–60%	28.091	0.349	<0.001	<0.001	0.089 [0.051; 0.126]	<0.001	0.130 [0.094; 0.165]	<0.001	0.041 [0.0001; 0.082]	0.148
ISO	Forceps major	60–80%	4.703	0.082	0.011	0.012	0.021 [-0.034; 0.076]	1.000	0.080 [0.028; 0.132]	0.009	0.059 [-0.0007; 0.119]	0.159
ISO	Forceps minor	20–40%	5.665	0.097	0.005	0.006	0.032 [-0.004; 0.069]	0.250	0.058 [0.023; 0.092]	0.004	0.026 [-0.014; 0.066]	0.622
ISO	Forceps minor	40–60%	9.126	0.148	<0.001	<0.001	0.047 [0.008; 0.086]	0.058	0.078 [0.041; 0.115]	<0.001	0.031 [-0.012; 0.074]	0.463
ISO	Forceps minor	60–80%	5.477	0.094	0.006	0.006	0.017 [-0.021; 0.055]	1.000	0.060 [0.024; 0.096]	0.004	0.043 [0.001; 0.084]	0.134
INTRA	Body	20–40%	68.481	0.585	<0.001	<0.001	-0.104 [-0.142; -0.066]	<0.001	-0.209 [-0.244; -0.173]	<0.001	-0.105 [-0.144; -0.065]	<0.001
INTRA	Body	40–60%	26.895	0.357	<0.001	<0.001	-0.100 [-0.139; -0.061]	<0.001	-0.128 [-0.165; -0.092]	<0.001	-0.028 [-0.069; 0.013]	0.514
INTRA	Body	60–80%	106.958	0.688	<0.001	<0.001	-0.119 [-0.155; -0.083]	<0.001	-0.245 [-0.279; -0.212]	<0.001	-0.127 [-0.164; -0.089]	<0.001
INTRA	Forceps major	20–40%	34.705	0.398	<0.001	<0.001	-0.093 [-0.138; -0.047]	<0.001	-0.182 [-0.226; -0.139]	<0.001	-0.090 [-0.140; -0.040]	0.002
INTRA	Forceps major	40–60%	52.131	0.498	<0.001	<0.001	-0.146 [-0.190; -0.102]	<0.001	-0.207 [-0.249; -0.165]	<0.001	-0.061 [-0.109; -0.013]	0.041
INTRA	Forceps major	60–80%	50.056	0.488	<0.001	<0.001	-0.123 [-0.171; -0.075]	<0.001	-0.227 [-0.272; -0.181]	<0.001	-0.104 [-0.156; -0.052]	<0.001
INTRA	Forceps minor	20–40%	38.796	0.425	<0.001	<0.001	-0.095 [-0.139; -0.051]	<0.001	-0.182 [-0.223; -0.141]	<0.001	-0.087 [-0.135; -0.039]	0.002

INTRA	Forceps minor	40–60%	40.356	0.435	<0.001	<0.001	-0.111 [-0.155; -0.067]	<0.001	-0.183 [-0.224; -0.142]	<0.001	-0.072 [-0.120; -0.024]	0.011
INTRA	Forceps minor	60–80%	29.754	0.362	<0.001	<0.001	-0.092 [-0.139; -0.044]	<0.001	-0.173 [-0.218; -0.128]	<0.001	-0.081 [-0.133; -0.029]	0.007
EXTRA_MD	Body	20–40%	34.081	0.413	<0.001	<0.001	0.065 [-0.012; 0.143]	0.290	0.293 [0.220; 0.365]	<0.001	0.227 [0.147; 0.308]	<0.001
EXTRA_MD	Body	40–60%	18.537	0.277	<0.001	<0.001	0.131 [0.054; 0.209]	0.004	0.221 [0.148; 0.294]	<0.001	0.090 [0.009; 0.172]	0.089
EXTRA_MD	Body	60–80%	68.483	0.585	<0.001	<0.001	0.116 [0.059; 0.174]	<0.001	0.315 [0.261; 0.369]	<0.001	0.199 [0.139; 0.259]	<0.001
EXTRA_MD	Forceps major	20–40%	19.381	0.270	<0.001	<0.001	0.143 [0.039; 0.246]	0.022	0.309 [0.210; 0.408]	<0.001	0.166 [0.053; 0.279]	0.013
EXTRA_MD	Forceps major	40–60%	61.632	0.540	<0.001	<0.001	0.294 [0.208; 0.381]	<0.001	0.445 [0.363; 0.528]	<0.001	0.151 [0.056; 0.245]	0.006
EXTRA_MD	Forceps major	60–80%	17.048	0.245	<0.001	<0.001	0.121 [0.014; 0.229]	0.083	0.302 [0.200; 0.405]	<0.001	0.181 [0.064; 0.299]	0.008
EXTRA_MD	Forceps minor	20–40%	40.192	0.434	<0.001	<0.001	0.125 [0.064; 0.187]	<0.001	0.263 [0.205; 0.321]	<0.001	0.137 [0.070; 0.205]	<0.001
EXTRA_MD	Forceps minor	40–60%	38.339	0.422	<0.001	<0.001	0.164 [0.090; 0.237]	<0.001	0.303 [0.234; 0.373]	<0.001	0.139 [0.059; 0.220]	0.002
EXTRA_MD	Forceps minor	60–80%	37.210	0.415	<0.001	<0.001	0.089 [0.030; 0.148]	0.011	0.243 [0.187; 0.299]	<0.001	0.154 [0.089; 0.219]	<0.001
EXTRA_TRANS	Body	20–40%	55.390	0.533	<0.001	<0.001	0.170 [0.076; 0.263]	0.002	0.461 [0.374; 0.549]	<0.001	0.291 [0.194; 0.389]	<0.001
EXTRA_TRANS	Body	40–60%	24.581	0.336	<0.001	<0.001	0.237 [0.130; 0.344]	<0.001	0.342 [0.242; 0.442]	<0.001	0.105 [-0.007; 0.216]	0.195
EXTRA_TRANS	Body	60–80%	103.683	0.681	<0.001	<0.001	0.228 [0.150; 0.306]	<0.001	0.529 [0.456; 0.602]	<0.001	0.301 [0.220; 0.382]	<0.001
EXTRA_TRANS	Forceps major	20–40%	27.655	0.345	<0.001	<0.001	0.227 [0.098; 0.355]	0.002	0.458 [0.335; 0.580]	<0.001	0.231 [0.091; 0.372]	0.004
EXTRA_TRANS	Forceps major	40–60%	59.830	0.533	<0.001	<0.001	0.414 [0.296; 0.532]	<0.001	0.594 [0.481; 0.707]	<0.001	0.180 [0.051; 0.309]	0.020

EXTRA_TRANS	Forceps major	60–80%	31.163	0.372	<0.001	<0.001	0.240 [0.107; 0.373]	0.002	0.504 [0.377; 0.631]	<0.001	0.264 [0.118; 0.409]	0.002
EXTRA_TRANS	Forceps minor	20–40%	57.118	0.521	<0.001	<0.001	0.212 [0.130; 0.294]	<0.001	0.414 [0.336; 0.491]	<0.001	0.202 [0.112; 0.291]	<0.001
EXTRA_TRANS	Forceps minor	40–60%	46.064	0.467	<0.001	<0.001	0.269 [0.166; 0.372]	<0.001	0.462 [0.365; 0.560]	<0.001	0.194 [0.081; 0.306]	0.003
EXTRA_TRANS	Forceps minor	60–80%	56.332	0.518	<0.001	<0.001	0.178 [0.102; 0.255]	<0.001	0.385 [0.313; 0.457]	<0.001	0.207 [0.124; 0.291]	<0.001
MK	Body	20–40%	73.680	0.603	<0.001	<0.001	-0.106 [-0.145; -0.067]	<0.001	-0.223 [-0.259; -0.186]	<0.001	-0.117 [-0.157; -0.076]	<0.001
MK	Body	40–60%	28.610	0.371	<0.001	<0.001	-0.115 [-0.161; -0.070]	<0.001	-0.156 [-0.199; -0.113]	<0.001	-0.041 [-0.089; 0.007]	0.280
MK	Body	60–80%	116.847	0.707	<0.001	<0.001	-0.119 [-0.155; -0.082]	<0.001	-0.261 [-0.294; -0.227]	<0.001	-0.142 [-0.180; -0.104]	<0.001
MK	Forceps major	20–40%	33.317	0.388	<0.001	<0.001	-0.103 [-0.153; -0.053]	<0.001	-0.194 [-0.241; -0.146]	<0.001	-0.091 [-0.145; -0.037]	0.004
MK	Forceps major	40–60%	32.660	0.384	<0.001	<0.001	-0.238 [-0.325; -0.151]	<0.001	-0.318 [-0.402; -0.235]	<0.001	-0.081 [-0.176; 0.014]	0.287
MK	Forceps major	60–80%	46.614	0.470	<0.001	<0.001	-0.149 [-0.205; -0.094]	<0.001	-0.254 [-0.307; -0.201]	<0.001	-0.104 [-0.165; -0.044]	0.003
MK	Forceps minor	20–40%	35.868	0.406	<0.001	<0.001	-0.098 [-0.149; -0.047]	<0.001	-0.207 [-0.255; -0.158]	<0.001	-0.109 [-0.165; -0.053]	<0.001
MK	Forceps minor	40–60%	29.163	0.357	<0.001	<0.001	-0.116 [-0.178; -0.054]	<0.001	-0.224 [-0.282; -0.165]	<0.001	-0.108 [-0.175; -0.040]	0.006
MK	Forceps minor	60–80%	32.753	0.384	<0.001	<0.001	-0.089 [-0.136; -0.042]	<0.001	-0.179 [-0.223; -0.135]	<0.001	-0.090 [-0.141; -0.039]	0.002
AK	Body	20–40%	64.393	0.570	<0.001	<0.001	-0.042 [-0.064; -0.020]	<0.001	-0.118 [-0.138; -0.097]	<0.001	-0.075 [-0.098; -0.052]	<0.001
AK	Body	40–60%	0.103	0.002	0.902	0.909	-0.003 [-0.024; 0.017]	1.000	-0.004 [-0.023; 0.015]	1.000	-0.0007 [-0.022; 0.020]	1.000
AK	Body	60–80%	64.912	0.572	<0.001	<0.001	-0.066 [-0.090; -0.042]	<0.001	-0.127 [-0.150; -0.105]	<0.001	-0.061 [-0.086; -0.036]	<0.001

AK	Forceps major	20–40%	6.053	0.103	0.003	0.004	-0.030 [-0.056; -0.003]	0.080	-0.042 [-0.066; -0.017]	0.004	-0.012 [-0.040; 0.017]	1.000
AK	Forceps major	40–60%	6.341	0.108	0.002	0.003	-0.049 [-0.078; -0.021]	0.003	-0.030 [-0.058; -0.003]	0.090	0.019 [-0.012; 0.050]	0.690
AK	Forceps major	60–80%	5.571	0.096	0.005	0.006	-0.036 [-0.068; -0.004]	0.090	-0.049 [-0.079; -0.018]	0.006	-0.013 [-0.048; 0.022]	1.000
AK	Forceps minor	20–40%	15.120	0.224	<0.001	<0.001	-0.053 [-0.084; -0.022]	0.003	-0.079 [-0.108; -0.049]	<0.001	-0.026 [-0.060; 0.008]	0.417
AK	Forceps minor	40–60%	4.897	0.085	0.009	0.010	-0.038 [-0.067; -0.010]	0.027	-0.034 [-0.061; -0.007]	0.040	0.004 [-0.027; 0.035]	1.000
AK	Forceps minor	60–80%	13.686	0.207	<0.001	<0.001	-0.046 [-0.080; -0.013]	0.022	-0.082 [-0.114; -0.051]	<0.001	-0.036 [-0.073; 0.0007]	0.163
RK	Body	20–40%	46.307	0.488	<0.001	<0.001	-0.205 [-0.295; -0.114]	<0.001	-0.411 [-0.495; -0.326]	<0.001	-0.206 [-0.301; -0.111]	<0.001
RK	Body	40–60%	22.801	0.320	<0.001	<0.001	-0.305 [-0.446; -0.165]	<0.001	-0.431 [-0.562; -0.300]	<0.001	-0.125 [-0.272; 0.021]	0.278
RK	Body	60–80%	90.868	0.652	<0.001	<0.001	-0.193 [-0.270; -0.116]	<0.001	-0.487 [-0.559; -0.415]	<0.001	-0.294 [-0.374; -0.214]	<0.001
RK	Forceps major	20–40%	21.293	0.289	<0.001	<0.001	-0.211 [-0.333; -0.089]	0.003	-0.379 [-0.496; -0.262]	<0.001	-0.168 [-0.302; -0.035]	0.042
RK	Forceps major	40–60%	44.412	0.458	<0.001	<0.001	-0.392 [-0.524; -0.260]	<0.001	-0.577 [-0.703; -0.450]	<0.001	-0.185 [-0.330; -0.040]	0.039
RK	Forceps major	60–80%	40.948	0.438	<0.001	<0.001	-0.300 [-0.425; -0.174]	<0.001	-0.541 [-0.661; -0.421]	<0.001	-0.242 [-0.379; -0.104]	0.002
RK	Forceps minor	20–40%	41.686	0.443	<0.001	<0.001	-0.144 [-0.229; -0.060]	0.003	-0.368 [-0.447; -0.288]	<0.001	-0.223 [-0.315; -0.131]	<0.001
RK	Forceps minor	40–60%	43.638	0.454	<0.001	<0.001	-0.224 [-0.345; -0.103]	0.001	-0.538 [-0.652; -0.424]	<0.001	-0.314 [-0.446; -0.182]	<0.001
RK	Forceps minor	60–80%	39.970	0.432	<0.001	<0.001	-0.133 [-0.207; -0.059]	0.002	-0.317 [-0.387; -0.246]	<0.001	-0.184 [-0.265; -0.102]	<0.001
AWF	Body	20–40%	70.038	0.591	<0.001	<0.001	-0.044 [-0.060; -0.027]	<0.001	-0.093 [-0.108; -0.077]	<0.001	-0.049 [-0.067; -0.032]	<0.001

AWF	Body	40–60%	29.346	0.377	<0.001	<0.001	-0.054 [-0.076; -0.031]	<0.001	-0.078 [-0.099; -0.058]	<0.001	-0.025 [-0.048; -0.001]	0.113
AWF	Body	60–80%	117.691	0.708	<0.001	<0.001	-0.046 [-0.060; -0.031]	<0.001	-0.106 [-0.120; -0.092]	<0.001	-0.060 [-0.076; -0.045]	<0.001
AWF	Forceps major	20–40%	34.410	0.396	<0.001	<0.001	-0.044 [-0.067; -0.020]	0.001	-0.094 [-0.116; -0.071]	<0.001	-0.050 [-0.075; -0.024]	<0.001
AWF	Forceps major	40–60%	47.182	0.473	<0.001	<0.001	-0.086 [-0.115; -0.057]	<0.001	-0.133 [-0.160; -0.105]	<0.001	-0.047 [-0.079; -0.015]	0.014
AWF	Forceps major	60–80%	52.713	0.501	<0.001	<0.001	-0.059 [-0.083; -0.035]	<0.001	-0.118 [-0.141; -0.095]	<0.001	-0.059 [-0.085; -0.032]	<0.001
AWF	Forceps minor	20–40%	57.499	0.523	<0.001	<0.001	-0.036 [-0.054; -0.019]	<0.001	-0.088 [-0.105; -0.072]	<0.001	-0.052 [-0.071; -0.033]	<0.001
AWF	Forceps minor	40–60%	52.209	0.499	<0.001	<0.001	-0.051 [-0.073; -0.028]	<0.001	-0.110 [-0.131; -0.088]	<0.001	-0.059 [-0.084; -0.034]	<0.001
AWF	Forceps minor	60–80%	69.431	0.569	<0.001	<0.001	-0.034 [-0.048; -0.020]	<0.001	-0.079 [-0.093; -0.066]	<0.001	-0.045 [-0.061; -0.030]	<0.001
axEAD	Body	20–40%	8.097	0.143	<0.001	<0.001	0.003 [-0.076; 0.082]	1.000	0.137 [0.063; 0.211]	0.001	0.134 [0.052; 0.217]	0.005
axEAD	Body	40–60%	4.605	0.087	0.012	0.014	-0.083 [-0.175; 0.009]	0.227	-0.129 [-0.215; -0.043]	0.011	-0.046 [-0.141; 0.050]	1.000
axEAD	Body	60–80%	12.148	0.200	<0.001	<0.001	0.092 [0.024; 0.161]	0.026	0.157 [0.093; 0.221]	<0.001	0.065 [-0.006; 0.136]	0.222
axEAD	Forceps major	20–40%	0.897	0.017	0.411	0.420	-0.057 [-0.191; 0.077]	1.000	0.042 [-0.087; 0.171]	1.000	0.099 [-0.048; 0.247]	0.553
axEAD	Forceps major	40–60%	0.227	0.005	0.797	0.808	-0.072 [-0.298; 0.153]	1.000	-0.046 [-0.259; 0.167]	1.000	0.026 [-0.223; 0.276]	1.000
axEAD	Forceps major	60–80%	1.054	0.021	0.352	0.362	-0.123 [-0.298; 0.051]	0.491	-0.067 [-0.227; 0.092]	1.000	0.056 [-0.135; 0.247]	1.000
axEAD	Forceps minor	20–40%	4.984	0.087	0.009	0.010	0.076 [0.016; 0.136]	0.039	0.078 [0.021; 0.135]	0.025	0.002 [-0.065; 0.068]	1.000
axEAD	Forceps minor	40–60%	4.411	0.078	0.014	0.016	0.031 [-0.051; 0.112]	1.000	-0.095 [-0.173; -0.017]	0.054	-0.126 [-0.216; -0.035]	0.020

axEAD	Forceps minor	60–80%	6.126	0.104	0.003	0.004	0.061 [-0.003; 0.125]	0.181	0.105 [0.044; 0.165]	0.003	0.043 [-0.027; 0.113]	0.664
radEAD	Body	20–40%	40.939	0.458	<0.001	<0.001	0.138 [0.057; 0.218]	0.003	0.341 [0.266; 0.416]	<0.001	0.204 [0.120; 0.287]	<0.001
radEAD	Body	40–60%	20.511	0.297	<0.001	<0.001	0.173 [0.093; 0.253]	<0.001	0.231 [0.156; 0.306]	<0.001	0.058 [-0.025; 0.142]	0.508
radEAD	Body	60–80%	84.328	0.635	<0.001	<0.001	0.181 [0.120; 0.242]	<0.001	0.371 [0.314; 0.428]	<0.001	0.190 [0.127; 0.254]	<0.001
radEAD	Forceps major	20–40%	9.653	0.157	<0.001	<0.001	0.099 [-0.053; 0.251]	0.601	0.323 [0.177; 0.469]	<0.001	0.224 [0.056; 0.391]	0.028
radEAD	Forceps major	40–60%	5.487	0.108	0.006	0.006	0.199 [0.021; 0.377]	0.086	0.255 [0.090; 0.420]	0.008	0.056 [-0.141; 0.252]	1.000
radEAD	Forceps major	60–80%	9.515	0.155	<0.001	<0.001	0.088 [-0.069; 0.246]	0.809	0.322 [0.174; 0.469]	<0.001	0.233 [0.061; 0.405]	0.025
radEAD	Forceps minor	20–40%	26.398	0.335	<0.001	<0.001	0.148 [0.074; 0.222]	<0.001	0.250 [0.181; 0.320]	<0.001	0.102 [0.022; 0.183]	0.041
radEAD	Forceps minor	40–60%	26.545	0.338	<0.001	<0.001	0.189 [0.102; 0.276]	<0.001	0.296 [0.213; 0.380]	<0.001	0.107 [0.011; 0.203]	0.087
radEAD	Forceps minor	60–80%	48.221	0.479	<0.001	<0.001	0.124 [0.067; 0.181]	<0.001	0.267 [0.213; 0.322]	<0.001	0.143 [0.081; 0.206]	<0.001

Note. FDR q refers to correction for multiple omnibus GLM comparisons; p Bonf. refers to pairwise post-hoc comparisons within the corresponding model. AD, MD, RD, axEAD, and radEAD, EXTRA_MD and EXTRA_TRANS are expressed in $\times 10^{-3}$ mm^2/s; . All other metrics are dimensionless.

Table S4. Stability of feature selection in the primary Elastic Net model

Feature	Number of outer CV iterations (out of 50	Selection frequency
NDI_CC_Body_5_95	50	100.0%
MK_CC_ForcepsMajor_5_95	49	98.0%
INTRA_CC_Body_5_95	45	90.0%
INTRA_CC_ForcepsMajor_5_95	44	88.0%
RK_CC_ForcepsMajor_5_95	44	88.0%
Age	44	88.0%
radEAD_CC_ForcepsMinor_5_95	42	84.0%
AWF_CC_ForcepsMajor_5_95	41	82.0%
RD_CC_ForcepsMinor_5_95	38	76.0%
EXTRA_TRANS_CC_ForcepsMinor_5_95	37	74.0%
FA_CC_ForcepsMinor_5_95	35	70.0%

MK_CC_Body_5_95	34	68.0%
MD_CC_ForcepsMinor_5_95	31	62.0%
AWF_CC_Body_5_95	29	58.0%
AK_CC_Body_5_95	26	52.0%
NDI_CC_ForcepsMajor_5_95	24	48.0%
FA_CC_ForcepsMajor_5_95	20	40.0%
EXTRA_MD_CC_ForcepsMinor_5_95	18	36.0%
RK_CC_Body_5_95	18	36.0%
EXTRA_TRANS_CC_ForcepsMajor_5_95	17	34.0%
ISO_CC_Body_5_95	15	30.0%
AWF_CC_ForcepsMinor_5_95	14	28.0%
ODI_CC_Body_5_95	14	28.0%
ODI_CC_ForcepsMajor_5_95	13	26.0%
FA_CC_Body_5_95	13	26.0%
ODI_CC_ForcepsMinor_5_95	11	22.0%
EXTRA_TRANS_CC_Body_5_95	11	22.0%
INTRA_CC_ForcepsMinor_5_95	8	16.0%
Sex	6	12.0%
AK_CC_ForcepsMajor_5_95	6	12.0%
axEAD_CC_ForcepsMinor_5_95	6	12.0%
ISO_CC_ForcepsMinor_5_95	5	10.0%
axEAD_CC_ForcepsMajor_5_95	4	8.0%
MK_CC_ForcepsMinor_5_95	4	8.0%
axEAD_CC_Body_5_95	3	6.0%
AK_CC_ForcepsMinor_5_95	3	6.0%
AD_CC_Body_5_95	3	6.0%
MD_CC_Body_5_95	3	6.0%
RD_CC_Body_5_95	2	4.0%
AD_CC_ForcepsMinor_5_95	2	4.0%
ISO_CC_ForcepsMajor_5_95	2	4.0%
EXTRA_MD_CC_ForcepsMajor_5_95	2	4.0%
radEAD_CC_ForcepsMajor_5_95	2	4.0%
RK_CC_ForcepsMinor_5_95	2	4.0%
AD_CC_ForcepsMajor_5_95	1	2.0%
RD_CC_ForcepsMajor_5_95	1	2.0%
radEAD_CC_Body_5_95	1	2.0%
NDI_CC_ForcepsMinor_5_95	1	2.0%
MD_CC_ForcepsMajor_5_95	0	0.0%
EXTRA_MD_Body_5_95	0	0.0%

Note. Selection frequency was calculated across 50 outer CV iterations (5 folds × 10 repeats).

Table S5. Summary comparison of MRI Type 1 and MRI Type 2 for models retaining an overall effect after FDR correction

Metrics	CC part	Interval	MRI Type 1 – MRI Type 2 [95% CI]	p Bonferroni

FA	Forceps major	5–95%	-0.059 [-0.087; -0.032]	<0.001
FA	Forceps minor	5–95%	-0.043 [-0.065; -0.022]	<0.001
FA	Body	5–95%	-0.060 [-0.080; -0.039]	<0.001
AD	Forceps major	5–95%	0.081 [0.006; 0.156]	0.103
AD	Forceps minor	5–95%	0.082 [0.035; 0.128]	0.002
AD	Body	5–95%	0.057 [0.017; 0.097]	0.018
MD	Forceps major	5–95%	0.121 [0.052; 0.190]	0.002
MD	Forceps minor	5–95%	0.103 [0.059; 0.148]	<0.001
MD	Body	5–95%	0.107 [0.064; 0.149]	<0.001
RD	Forceps major	5–95%	0.141 [0.066; 0.216]	<0.001
RD	Forceps minor	5–95%	0.115 [0.065; 0.165]	<0.001
RD	Body	5–95%	0.133 [0.084; 0.182]	<0.001
NDI	Forceps major	5–95%	-0.069 [-0.102; -0.036]	<0.001
NDI	Forceps minor	5–95%	-0.057 [-0.110; -0.005]	0.095
NDI	Body	5–95%	-0.089 [-0.119; -0.059]	<0.001
ODI	Forceps major	5–95%	0.010 [-0.002; 0.023]	0.276
ODI	Body	5–95%	0.010 [-0.002; 0.021]	0.306
ISO	Forceps major	5–95%	0.028 [-0.009; 0.065]	0.408
ISO	Forceps minor	5–95%	0.023 [-0.011; 0.057]	0.570
ISO	Body	5–95%	0.006 [-0.021; 0.033]	1.000
INTRA	Forceps major	5–95%	-0.099 [-0.132; -0.066]	<0.001
INTRA	Forceps minor	5–95%	-0.076 [-0.117; -0.036]	<0.001
INTRA	Body	5–95%	-0.094 [-0.123; -0.066]	<0.001
EXTRA_MD	Forceps major	5–95%	0.114 [0.044; 0.183]	0.005
EXTRA_MD	Forceps minor	5–95%	0.097 [0.045; 0.149]	0.001
EXTRA_MD	Body	5–95%	0.075 [0.024; 0.125]	0.014
EXTRA_TRANS	Forceps major	5–95%	0.207 [0.121; 0.294]	<0.001
EXTRA_TRANS	Forceps minor	5–95%	0.170 [0.103; 0.237]	<0.001
EXTRA_TRANS	Body	5–95%	0.172 [0.109; 0.236]	<0.001
MK	Forceps major	5–95%	-0.128 [-0.169; -0.087]	<0.001
MK	Forceps minor	5–95%	-0.079 [-0.124; -0.033]	0.003
MK	Body	5–95%	-0.096 [-0.127; -0.065]	<0.001
AK	Forceps major	5–95%	-0.032 [-0.055; -0.009]	0.023
AK	Forceps minor	5–95%	-0.039 [-0.069; -0.008]	0.042
AK	Body	5–95%	-0.037 [-0.054; -0.021]	<0.001
RK	Forceps major	5–95%	-0.234 [-0.316; -0.152]	<0.001
RK	Forceps minor	5–95%	-0.124 [-0.192; -0.055]	0.002
RK	Body	5–95%	-0.191 [-0.263; -0.118]	<0.001
AWF	Forceps major	5–95%	-0.048 [-0.064; -0.031]	<0.001

AWF	Forceps minor	5–95%	-0.030 [-0.044; -0.017]	<0.001
AWF	Body	5–95%	-0.040 [-0.053; -0.027]	<0.001
axEAD	Forceps minor	5–95%	0.050 [0.004; 0.095]	0.100
axEAD	Body	5–95%	0.006 [-0.038; 0.050]	1.000
radEAD	Forceps minor	5–95%	0.116 [0.058; 0.174]	<0.001
radEAD	Body	5–95%	0.128 [0.076; 0.179]	<0.001
FA	Body	20–40%	-0.064 [-0.092; -0.037]	<0.001
FA	Body	40–60%	-0.094 [-0.132; -0.055]	<0.001
FA	Body	60–80%	-0.063 [-0.086; -0.039]	<0.001
FA	Forceps major	20–40%	-0.065 [-0.105; -0.025]	0.005
FA	Forceps major	40–60%	-0.086 [-0.127; -0.046]	<0.001
FA	Forceps major	60–80%	-0.075 [-0.115; -0.035]	<0.001
FA	Forceps minor	20–40%	-0.053 [-0.082; -0.025]	<0.001
FA	Forceps minor	40–60%	-0.078 [-0.116; -0.039]	<0.001
FA	Forceps minor	60–80%	-0.045 [-0.067; -0.023]	<0.001
AD	Body	20–40%	0.057 [-0.017; 0.132]	0.392
AD	Body	60–80%	0.136 [0.072; 0.200]	<0.001
AD	Forceps major	20–40%	0.102 [-0.007; 0.211]	0.202
AD	Forceps major	40–60%	0.202 [0.114; 0.290]	<0.001
AD	Forceps major	60–80%	0.088 [-0.033; 0.208]	0.457
AD	Forceps minor	20–40%	0.110 [0.052; 0.169]	<0.001
AD	Forceps minor	40–60%	0.098 [0.023; 0.172]	0.034
AD	Forceps minor	60–80%	0.094 [0.032; 0.156]	0.010
MD	Body	20–40%	0.115 [0.044; 0.186]	0.005
MD	Body	40–60%	0.115 [0.052; 0.177]	0.001
MD	Body	60–80%	0.167 [0.111; 0.222]	<0.001
MD	Forceps major	20–40%	0.146 [0.042; 0.251]	0.019
MD	Forceps major	40–60%	0.218 [0.147; 0.289]	<0.001
MD	Forceps major	60–80%	0.144 [0.038; 0.251]	0.025
MD	Forceps minor	20–40%	0.134 [0.078; 0.189]	<0.001
MD	Forceps minor	40–60%	0.152 [0.085; 0.219]	<0.001
MD	Forceps minor	60–80%	0.113 [0.061; 0.166]	<0.001
RD	Body	20–40%	0.146 [0.070; 0.223]	<0.001
RD	Body	40–60%	0.172 [0.095; 0.249]	<0.001
RD	Body	60–80%	0.184 [0.124; 0.244]	<0.001
RD	Forceps major	20–40%	0.170 [0.055; 0.284]	0.012
RD	Forceps major	40–60%	0.226 [0.143; 0.309]	<0.001
RD	Forceps major	60–80%	0.172 [0.059; 0.286]	0.010
RD	Forceps minor	20–40%	0.146 [0.082; 0.209]	<0.001

RD	Forceps minor	40–60%	0.180 [0.099; 0.261]	<0.001
RD	Forceps minor	60–80%	0.123 [0.069; 0.178]	<0.001
NDI	Body	20–40%	-0.109 [-0.150; -0.069]	<0.001
NDI	Body	40–60%	-0.090 [-0.127; -0.053]	<0.001
NDI	Body	60–80%	-0.092 [-0.127; -0.057]	<0.001
NDI	Forceps major	20–40%	-0.060 [-0.098; -0.022]	0.007
NDI	Forceps major	40–60%	-0.077 [-0.129; -0.025]	0.012
NDI	Forceps major	60–80%	-0.102 [-0.144; -0.059]	<0.001
NDI	Forceps minor	20–40%	-0.073 [-0.128; -0.019]	0.027
NDI	Forceps minor	40–60%	-0.066 [-0.123; -0.009]	0.070
NDI	Forceps minor	60–80%	-0.071 [-0.128; -0.014]	0.045
ODI	Body	40–60%	0.019 [0.004; 0.034]	0.038
ODI	Forceps minor	40–60%	0.018 [-0.003; 0.038]	0.256
ISO	Body	20–40%	-0.0005 [-0.043; 0.042]	1.000
ISO	Body	60–80%	0.031 [0.0007; 0.062]	0.135
ISO	Forceps major	20–40%	0.047 [-0.007; 0.100]	0.258
ISO	Forceps major	40–60%	0.089 [0.051; 0.126]	<0.001
ISO	Forceps major	60–80%	0.021 [-0.034; 0.076]	1.000
ISO	Forceps minor	20–40%	0.032 [-0.004; 0.069]	0.250
ISO	Forceps minor	40–60%	0.047 [0.008; 0.086]	0.058
ISO	Forceps minor	60–80%	0.017 [-0.021; 0.055]	1.000
INTRA	Body	20–40%	-0.104 [-0.142; -0.066]	<0.001
INTRA	Body	40–60%	-0.100 [-0.139; -0.061]	<0.001
INTRA	Body	60–80%	-0.119 [-0.155; -0.083]	<0.001
INTRA	Forceps major	20–40%	-0.093 [-0.138; -0.047]	<0.001
INTRA	Forceps major	40–60%	-0.146 [-0.190; -0.102]	<0.001
INTRA	Forceps major	60–80%	-0.123 [-0.171; -0.075]	<0.001
INTRA	Forceps minor	20–40%	-0.095 [-0.139; -0.051]	<0.001
INTRA	Forceps minor	40–60%	-0.111 [-0.155; -0.067]	<0.001
INTRA	Forceps minor	60–80%	-0.092 [-0.139; -0.044]	<0.001
EXTRA_MD	Body	20–40%	0.065 [-0.012; 0.143]	0.290
EXTRA_MD	Body	40–60%	0.131 [0.054; 0.209]	0.004
EXTRA_MD	Body	60–80%	0.116 [0.059; 0.174]	<0.001
EXTRA_MD	Forceps major	20–40%	0.143 [0.039; 0.246]	0.022
EXTRA_MD	Forceps major	40–60%	0.294 [0.208; 0.381]	<0.001
EXTRA_MD	Forceps major	60–80%	0.121 [0.014; 0.229]	0.083
EXTRA_MD	Forceps minor	20–40%	0.125 [0.064; 0.187]	<0.001
EXTRA_MD	Forceps minor	40–60%	0.164 [0.090; 0.237]	<0.001
EXTRA_MD	Forceps minor	60–80%	0.089 [0.030; 0.148]	0.011

EXTRA_TRANS	Body	20–40%	0.170 [0.076; 0.263]	0.002
EXTRA_TRANS	Body	40–60%	0.237 [0.130; 0.344]	<0.001
EXTRA_TRANS	Body	60–80%	0.228 [0.150; 0.306]	<0.001
EXTRA_TRANS	Forceps major	20–40%	0.227 [0.098; 0.355]	0.002
EXTRA_TRANS	Forceps major	40–60%	0.414 [0.296; 0.532]	<0.001
EXTRA_TRANS	Forceps major	60–80%	0.240 [0.107; 0.373]	0.002
EXTRA_TRANS	Forceps minor	20–40%	0.212 [0.130; 0.294]	<0.001
EXTRA_TRANS	Forceps minor	40–60%	0.269 [0.166; 0.372]	<0.001
EXTRA_TRANS	Forceps minor	60–80%	0.178 [0.102; 0.255]	<0.001
MK	Body	20–40%	-0.106 [-0.145; -0.067]	<0.001
MK	Body	40–60%	-0.115 [-0.161; -0.070]	<0.001
MK	Body	60–80%	-0.119 [-0.155; -0.082]	<0.001
MK	Forceps major	20–40%	-0.103 [-0.153; -0.053]	<0.001
MK	Forceps major	40–60%	-0.238 [-0.325; -0.151]	<0.001
MK	Forceps major	60–80%	-0.149 [-0.205; -0.094]	<0.001
MK	Forceps minor	20–40%	-0.098 [-0.149; -0.047]	<0.001
MK	Forceps minor	40–60%	-0.116 [-0.178; -0.054]	<0.001
MK	Forceps minor	60–80%	-0.089 [-0.136; -0.042]	<0.001
AK	Body	20–40%	-0.042 [-0.064; -0.020]	<0.001
AK	Body	60–80%	-0.066 [-0.090; -0.042]	<0.001
AK	Forceps major	20–40%	-0.030 [-0.056; -0.003]	0.080
AK	Forceps major	40–60%	-0.049 [-0.078; -0.021]	0.003
AK	Forceps major	60–80%	-0.036 [-0.068; -0.004]	0.090
AK	Forceps minor	20–40%	-0.053 [-0.084; -0.022]	0.003
AK	Forceps minor	40–60%	-0.038 [-0.067; -0.010]	0.027
AK	Forceps minor	60–80%	-0.046 [-0.080; -0.013]	0.022
RK	Body	20–40%	-0.205 [-0.295; -0.114]	<0.001
RK	Body	40–60%	-0.305 [-0.446; -0.165]	<0.001
RK	Body	60–80%	-0.193 [-0.270; -0.116]	<0.001
RK	Forceps major	20–40%	-0.211 [-0.333; -0.089]	0.003
RK	Forceps major	40–60%	-0.392 [-0.524; -0.260]	<0.001
RK	Forceps major	60–80%	-0.300 [-0.425; -0.174]	<0.001
RK	Forceps minor	20–40%	-0.144 [-0.229; -0.060]	0.003
RK	Forceps minor	40–60%	-0.224 [-0.345; -0.103]	0.001
RK	Forceps minor	60–80%	-0.133 [-0.207; -0.059]	0.002
AWF	Body	20–40%	-0.044 [-0.060; -0.027]	<0.001
AWF	Body	40–60%	-0.054 [-0.076; -0.031]	<0.001
AWF	Body	60–80%	-0.046 [-0.060; -0.031]	<0.001

AWF	Forceps major	20–40%	-0.044 [-0.067; -0.020]	0.001
AWF	Forceps major	40–60%	-0.086 [-0.115; -0.057]	<0.001
AWF	Forceps major	60–80%	-0.059 [-0.083; -0.035]	<0.001
AWF	Forceps minor	20–40%	-0.036 [-0.054; -0.019]	<0.001
AWF	Forceps minor	40–60%	-0.051 [-0.073; -0.028]	<0.001
AWF	Forceps minor	60–80%	-0.034 [-0.048; -0.020]	<0.001
axEAD	Body	20–40%	0.003 [-0.076; 0.082]	1.000
axEAD	Body	40–60%	-0.083 [-0.175; 0.009]	0.227
axEAD	Body	60–80%	0.092 [0.024; 0.161]	0.026
axEAD	Forceps minor	20–40%	0.076 [0.016; 0.136]	0.039
axEAD	Forceps minor	40–60%	0.031 [-0.051; 0.112]	1.000
axEAD	Forceps minor	60–80%	0.061 [-0.003; 0.125]	0.181
radEAD	Body	20–40%	0.138 [0.057; 0.218]	0.003
radEAD	Body	40–60%	0.173 [0.093; 0.253]	<0.001
radEAD	Body	60–80%	0.181 [0.120; 0.242]	<0.001
radEAD	Forceps major	20–40%	0.099 [-0.053; 0.251]	0.601
radEAD	Forceps major	40–60%	0.199 [0.021; 0.377]	0.086
radEAD	Forceps major	60–80%	0.088 [-0.069; 0.246]	0.809
radEAD	Forceps minor	20–40%	0.148 [0.074; 0.222]	<0.001
radEAD	Forceps minor	40–60%	0.189 [0.102; 0.276]	<0.001
radEAD	Forceps minor	60–80%	0.124 [0.067; 0.181]	<0.001

Note. p Bonferroni refers to the Bonferroni-adjusted p-value for the MRI Type 1 vs MRI Type 2 comparison. AD, MD, RD, axEAD, radEAD, EXTRA_MD and EXTRA_TRANS are expressed in $\times 10^{-3}$ mm^2/s. All other metrics are dimensionless.

Table S6. Generalized characteristics of SVD MRI types at Fazekas stage 3, based on the results of the present study and earlier studies by the author group [20–23].

Feature	MRI Type 1	MRI Type 2
Age	Younger	Older
Sex	Male predominance	Female predominance
Vascular risk factors	No differences	
Severity of CI	More pronounced	Less pronounced
Total SVD score	3–4 points	2–3 points
WMH [20]	Predominantly confluent WMH in the periventricular and deep white matter of the frontal and parietal lobes, extending into the deep white matter of the occipital lobe	Predominantly periventricular WMH in the posterior regions and deep WMH in the frontal and parietal lobes; mildly expressed WMH in subcortical structures
Lacunes	Multiple, in white matter and subcortical structures	Few, in white matter and subcortical structures
Cerebral microbleeds	Juxtacortical and subcortical	None juxtacortical or subcortical; few in the periventricular white

		matter of the temporal and occipital regions
Atrophy	More frequent; greater enlargement of CSF spaces	Less frequent
Enlarged perivascular spaces	More pronounced in subcortical structures (>2–3 mm in ½ of patients)	Less pronounced
Diffusion MRI (tract-profile analysis of corpus callosum segments)	Lower FA, NDI, INTRA, AWF, MK, AK, RK; higher MD, RD, EXTRA_TRANS	Higher FA, NDI, INTRA, AWF, MK, AK, RK; lower MD, RD, EXTRA_TRANS
Dynamic contrast-enhanced T1 MRI [21, 22]	Lower AUC in WMH, Vp in NAWM and WMH, K_{trans} in all regions	Higher AUC in WMH, Vp in NAWM and WMH, K_{trans} in all regions
Circulating blood markers of vascular wall and brain damage [23]	Decreased VEGF-A	Increased TNF-α

Abbreviations: CI – cognitive impairment; WMH – white matter hyperintensity; WM – white matter; Vp – fractional plasma volume; AUC – area under the curve; Ktrans – capillary permeability coefficient; GM – gray matter; NAWM – normal-appearing white matter; VEGF-A – vascular endothelial growth factor-A; TNF-α – tumor necrosis factor-alpha.
